# Predicting natural enemy efficacy in biological control using ex-ante analyses

**DOI:** 10.1038/s41598-025-29022-1

**Published:** 2025-12-29

**Authors:** Andrew Paul Gutierrez, Luigi Ponti, Peter Neuenschwander, John S. Yaninek, Hans R. Herren

**Affiliations:** 1 Center for the Analysis of Sustainable Agroecological Systems, Kensington, CA 94707-1035 USA; 2 Division of Ecosystem Science, College of Natural Resources, University of California, Berkeley, CA 94720-3114 USA; 3 Agenzia nazionale per le nuove tecnologie, l’energia e lo sviluppo economico sostenibile (ENEA), Centro Ricerche Casaccia, Via Anguillarese 301, 00123 Roma, Italy; 4 International Institute of Tropical Agriculture, Biological Control Center for Africa, 08 B.P. 0932, Cotonou, Benin; 5 Department of Entomology, Purdue University, West Lafayette, IN 47907-2089 USA; 6 The Millennium Institute, Washington, DC USA

**Keywords:** Biological control, Physiologically based demographic model, Time-varying life tables, Bioeconomic analysis, Ecology, Ecology, Plant sciences

## Abstract

Massive losses in agricultural and natural systems accrue globally due to invasive species, and yet the success rate of natural enemy introductions to control them is low. The high failure rate is due to the unknown efficacy of the introduced natural enemies. Furthermore, reviews of prior biological control efforts have not led to the development of assessment methods to predict their pre-release efficacy. To demonstrate a potential solution, we deconstructed the biological control of the invasive cassava mealybug (CM) and cassava green mite (CGM) in Africa using weather-driven metapopulation tri-trophic physiologically based demographic models (PBDMs). Bioeconomic analysis of the simulation results enabled parsing the contributions of the introduced natural enemies and endemic fungal pathogens to the control of CM and CGM and to the recovery of cassava yield across the vast ecological zones of Africa. The analysis shows that *ex-ante* pre-release analyses of natural enemy efficacy would have correctly predicted the biological control of the two pests. PBDM analyses of other biological control programs explained their success and/or failure. The results suggest well-parameterized mechanistic models can predict pre-release the efficacy of natural enemies and become an important instrument in increasing global food security.

**Supplementary Information:**

The online version contains supplementary material available at 10.1038/s41598-025-29022-1.

## Introduction

Massive losses occur globally due to invasive species that may disrupt extant food webs in novel environments. Classical biological control has a proven record of success in controlling invasive pests, with the control of cottony cushion scale (*Icerya purchasi* Maskell) in California citrus in 1888 by the introduced vedalia beetle (*Rodolia cardinalis* (Mulsant)) and the parasitic fly *Cryptochaetum iceryae* (Williston) being the hallmark example^1^. Since the late nineteenth century, more than 2000 natural enemy species (agents) have been released in programs against approximately 400 invasive species worldwide^2,3^, resulting in partial or complete control of 226 invasive insect and 57 invasive weed species, with only ten cases resulting in negative outcomes^4^. However, the rate of introductions slowed after 1990 when the perspective in biological control changed focus from benefit to risk assessment, highlighting the need for conceptual models to guide benefit-risk decisions^4^. But there is little evidence that particular biological traits of natural enemies consistently predict the success of biological control, a conclusion attributed to insufficient data^5^. In general, biological control is significantly higher in single-species releases against insect pests than in multiple-species releases, but this contrasts with weeds, where control increases with the number of species released^6^.

Theoretical differential models of classical biological control systems abound in the literature, with most concerning host-parasitoid interactions. These models have been used to make predictions about the independent effects of natural enemy attributes (e.g., sex ratio, reproductive strategies, fecundity, host feeding, developmental delays, dispersal rates, refuges, etc.) on system equilibrium with inference about control^7^. However, the predictions of such models are difficult to assess in the field, though some models have provided important theoretical insights^8,9^. The call to develop realistic field models to fill the gap between theory and practice has a long history^7,10–12^, with some^13^ calling for an inclusive theory of biological control that incorporates the demographic and genetic processes to address the establishment and impact of introduced natural enemies.

Field applications of models require incorporating the weather-driven, bottom-up effects of plants on higher trophic levels and the top-down effects of consumer behavior and physiology (sensu Hairston^14^). However, because of the myriad variant biology and complexity of species interactions, predicting the efficacy of natural enemy introductions has been assumed to be problematic^10^, but we demonstrate here that this need not be the case. Still, to determine what aspects of the biology of host-natural enemies are responsible for control or failure, we must be able to deconstruct holistically the weather-driven biology and dynamics of systems. Recent work demonstrated the ability to model the geospatial distribution and relative abundance of multiple invasive species^15^, but a critical gap remains: the ability to move from predicting where a pest or natural enemy might establish to predicting how well a specific biological control agent will perform before it is released. This study attempts to bridge some of this gap.

**Fig. 1:**
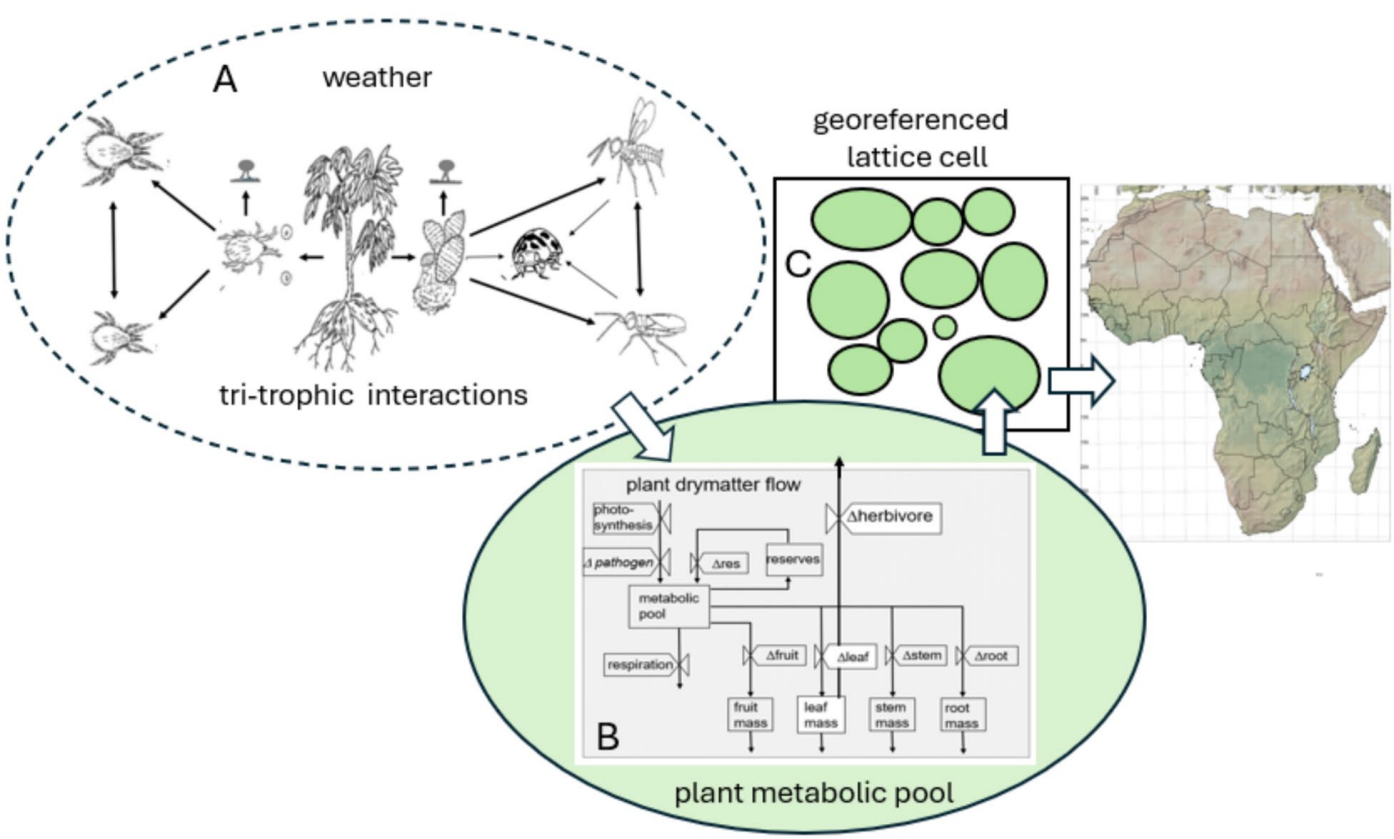
A physiologically based demographic tri-trophic meta-population cassava system for Africa: (**A**) tri-trophic species interactions on individual cassava plants where single arrows indicate the direction of dry matter flow, double arrows indicate intraspecific competition, and pathogens are indicated as small symbols for conidia above CM and CGM, (**B**) the within plant flow of dry matter to age structured subunit populations, to herbivores (i.e., CM and CGM) and to higher tropic levels, and (**C**) individual plant interactions with each plant having populations of the species depicted in 1A with movement of mobile arthropod stages between plants governed by species-specific resource supply/demand ratios^16^. The map for Africa was developed using the open source GRASS GIS^33^ and open access geospatial data^34^.

In practice, we must use post hoc analyses of past natural enemy introductions to assess what aspects of the biology were crucial to control or failure. To illustrate this process, we deconstruct the highly successful biological control of two pests of cassava (*Manihot esculenta* Crantz) on the local scale and across the vast ecological zones of Africa using tri-trophic physiologically based demographic models (PBDMs) of the species in the system (Fig. 1A)^16^. First, we provide historical overview of cassava, the two exotic pests and the endemic and introduced natural enemies. A brief review of the biology of the species is given in the text, with greater details reported in the cited references and in the Supplemental Materials. We then simulate the weather-driven dynamics of the system across Africa and conduct marginal bioeconomic analyses of the area-wide results to parse the contributions of the natural enemies to the control of the two pests and to yield recovery. The approach is applicable to any system^15^ and demonstrating this is a thrust of our study. Self-reference is unavoidable as much of the relevant literature on the biological control of cassava pests in Africa and the development of the physiologically based model structure is by the authors.

### Historical perspective of cassava in Africa

Cassava was introduced to Africa from Brazil during the slave trade in the sixteenth century, becoming an important staple crop across sub-Saharan Africa^17^. Two Neotropical pests, the cassava mealybug (*Phenacoccus manihoti* Matile-Ferrero (Hemiptera, Pseudococcidae)) (CM) and cassava green mite (*Mononychellus tanajoa* (Bondar), (Trombidiformes, Tetranychidae)) (CGM) were accidentally introduced to Africa early in the 1970s on cassava cuttings for use in plant breeding^18,19^. The two pests quickly spread across the African cassava zone causing massive yield losses creating severe food insecurity for more than 200 million Africans^20^.

In Africa, CM was attacked by native generalist ladybird beetle predators in the genera *Hyperaspis, Exochomus* and *Diomus*, but they had minimal impact^21,22^. CM was also infected by the endemic fungal pathogen (*Neozygites fumosa* (Zygomycetes, Entomophthorales))^23^, especially during the rainy season^24^. Foreign exploration for effective natural enemies was conducted in the centers of cassava origin in Central and South America that resulted in the introduction, among others, of two hymenopterous parasitoid wasps (*Anagyrus lopezi* DeSantis and *A. diversicornis* Howard) (Hymenoptera, Encyrtidae)^25^. Both parasitoids were widely released, but *A. diversicornis* appears to have failed beyond the initial release sites in Benin^26,27^, while *A. lopezi* established widely and controlled CM populations at low levels across Africa^28,29^ and in Asia where CM subsequently spread^30^.

CGM in Africa was attacked by native generalist insect predators and an endemic fungal pathogen, but with limited impact^18^. Eleven species of predators of CGM in the family Phytoseiidae were introduced from South America during the period 1984–2001, and more than 11.3 million were reared and released in 20 African countries^31^. Of these, only three of the predators established (*Neoseiulus idaeus* Denmark & Muma, *Amblydromalus manihoti* Moraes, and *Typhlodromalus aripo* De Leon), but only *A. manihoti* and *T. aripo* spread widely reducing CGM populations by half and increasing cassava yields by a third^31^. A virulent strain of the CGM-specific fungus *Neozygites tanajoae* was also introduced from Brazil to Benin, where local impact was observed^32^, but its spread was not monitored.

## Results

As a guide, we first simulate the weather driven dynamics of the system components across Africa and then conduct bioeconomic analyses of the results to parse the contributions of the natural enemies to yield recovery and to the control of the two pests.

### Cassava

**Fig. 2:**
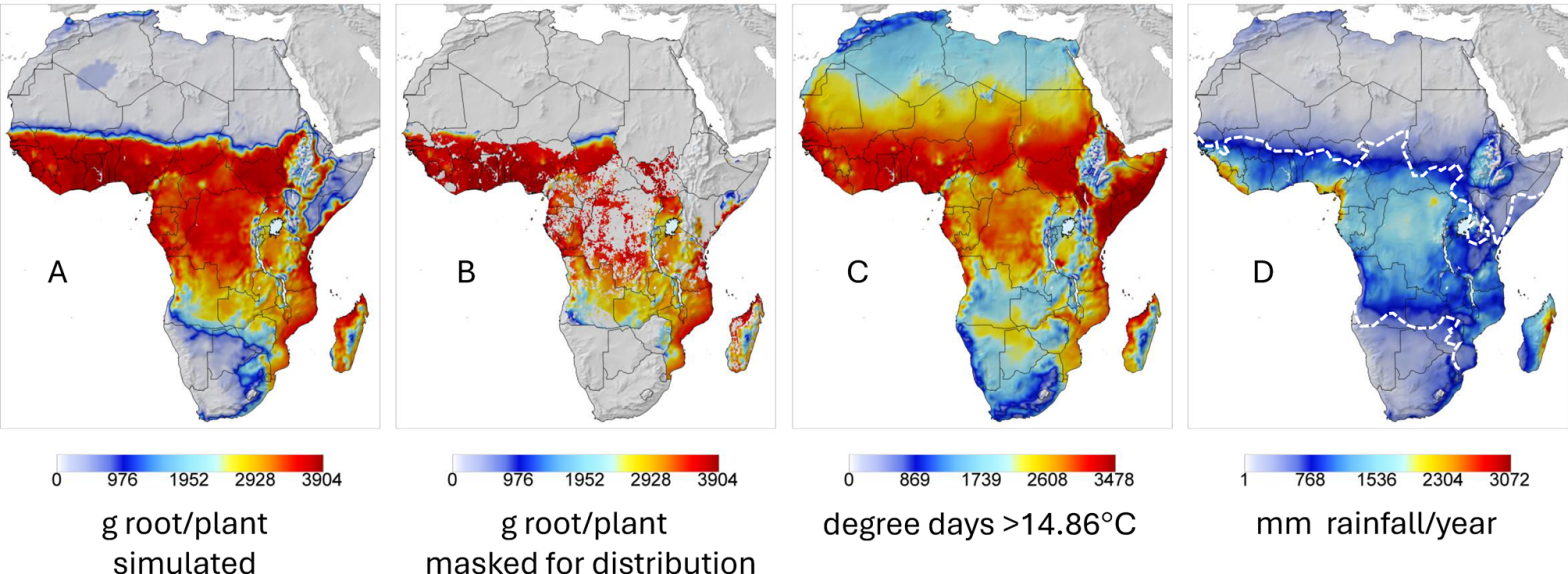
Simulated pest-free cassava in Africa during years 1981–1990: (**A**) prospective distribution and average root yield (g dry matter per plant), (**B**) root yield data from Fig. 2A masked for the known distribution of cassava^35^ (public repository: https://doi.org/10.6084/m9.figshare.22491997), (**C**) average annual degree days > 14.85 °C for cassava estimated using a nonlinear model (Supplemental Materials) based on cassava growth rates data^36^, and (**D**) mean annual mm of rainfall with the limits of cassava circumscribed by the dashed white lines^35^.

The prospective geographic distribution of average root yield per plant (grams) during the 1981–1990 period is mapped in Fig. 2A, while the same data masked for the historical distribution of cassava^35^ are mapped in Fig. 2B. Average annual degree days computed using a nonlinear model (*dd* > 14.86 °C) for cassava (Supplemental Materials) are mapped in Fig. 2C, and average annual rainfall is mapped in Fig. 2D with the limits of cassava production indicated by dashed lines^35^. The simulated distribution of cassava coincides well with the distribution of rainfall above the ~ 750 mm isohyet. The model predicts yields in South Sudan and parts of Uganda, Kenya, and Ethiopia (Fig. 2A vs. masked Fig. 2B), where alternative crops (e.g., maize, sorghum) are more prevalent. The gaps in Central Africa (Fig. 2B) are forested areas. All subsequent results are masked for the observed distribution of cassava^35^.

**Fig. 3:**
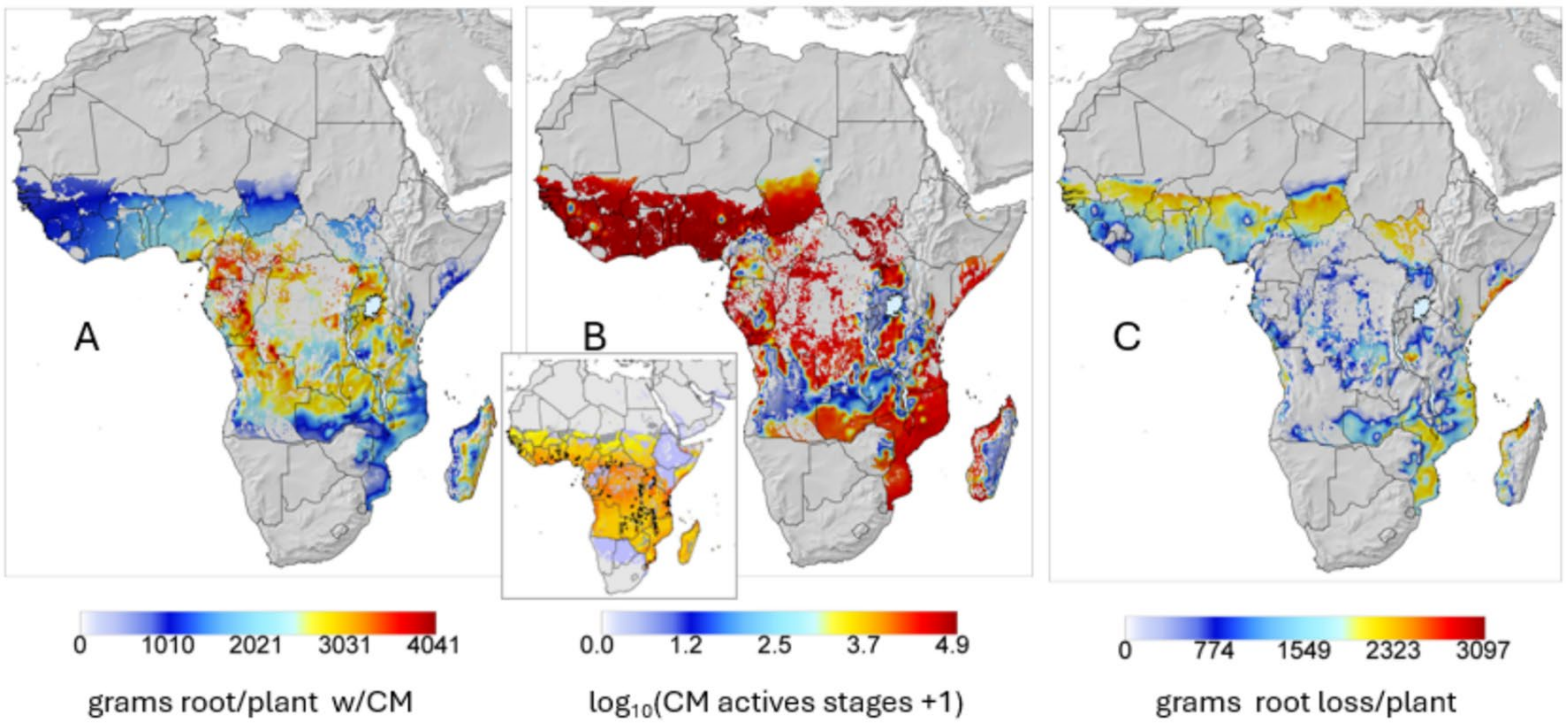
Prospective per plant averages over the 1981–1990 period before biological control of CM given the effect of the endemic fungal pathogen: (**A**) prospective distribution of cassava root yield (g dry matter per plant) after the invasion of the cassava mealybug (CM), (**B**) prospective distribution of log_10_ cumulative annual mealybug active stages per plant with an inset clip of the predicted CLIMEX distribution of CM^37^, and (**C**) yield loss per plant due to uncontrolled mealybug computed as the difference of yields in Fig. 2B minus yields in Fig. 3A. The small difference in the scales between Figs. 2B and 3A is due to GIS system interpolation.

*Impact of cassava mealybug on yield—*Absent natural control of CM, simulated average annual root yields (g dry matter per plant) over the 1981–1990 period are mapped in Fig. 3A. log_10_ average annual cumulative CM active stages per plant given the action of the endemic fungal pathogen are mapped in Fig. 3B. Prospective root yield losses are mapped in Fig. 3C as the difference of data in Figs. 2B and 3A. Model prediction of the geographic distribution of CM (Fig. 3B) agrees qualitatively with the distribution of CM estimated using the species distribution model CLIMEX^37^ (inset in Fig. 3B).

**Fig. 4:**
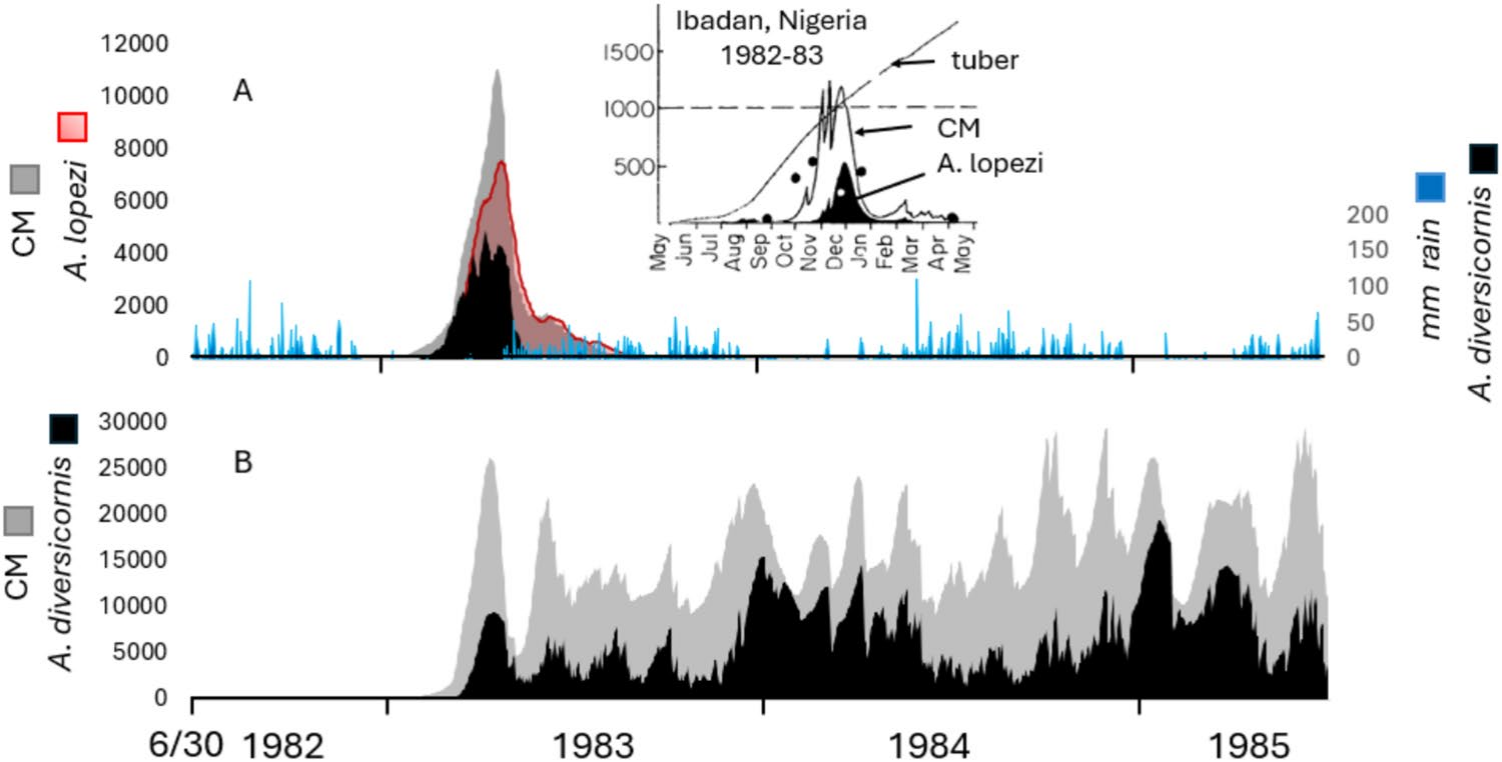
Simulated average per plant population dynamics at Cotonou, Benin during 6/30/1982 to 6/30/1985: (**A**) cassava mealybug (CM), and CM parasitized by *A. lopezi* and *A. diversicornis* given the impact of fungal pathogen mortality on CM, and (**B**) CM and *A. diversicornis* parasitized CM given fungal mortality. Daily rainfall is indicated as the blue lines in 4A, and the inset shows data from Ibadan, Nigeria^38^.

*Biological control of cassava mealybug*—Initial releases of the two parasitoids occurred at Cotonou, Benin, West Africa, and using Jun 30, 1982 to June 30, 1985 weather, rapid local control of CM by *A. lopezi*, *A. diversicornis* and the endemic fungal pathogen is predicted (Fig. 4A). Similar data for Ibadan, Nigeria are illustrated in the inset^38^. *A. lopezi* in concert with rainfall/fungal mortality suppress CM populations to very low levels with near total displacement of *A. diversicornis*^16^. However, absent *A. lopezi*, high populations of *A. diversicornis* are predicted but do not provide economic control (Fig. 4B). However, despite the minor role of *A. diversicornis* in suppressing CM locally^27^, it was included in the Africa-wide study to explore its potential in other ecological regions.

**Fig. 5:**
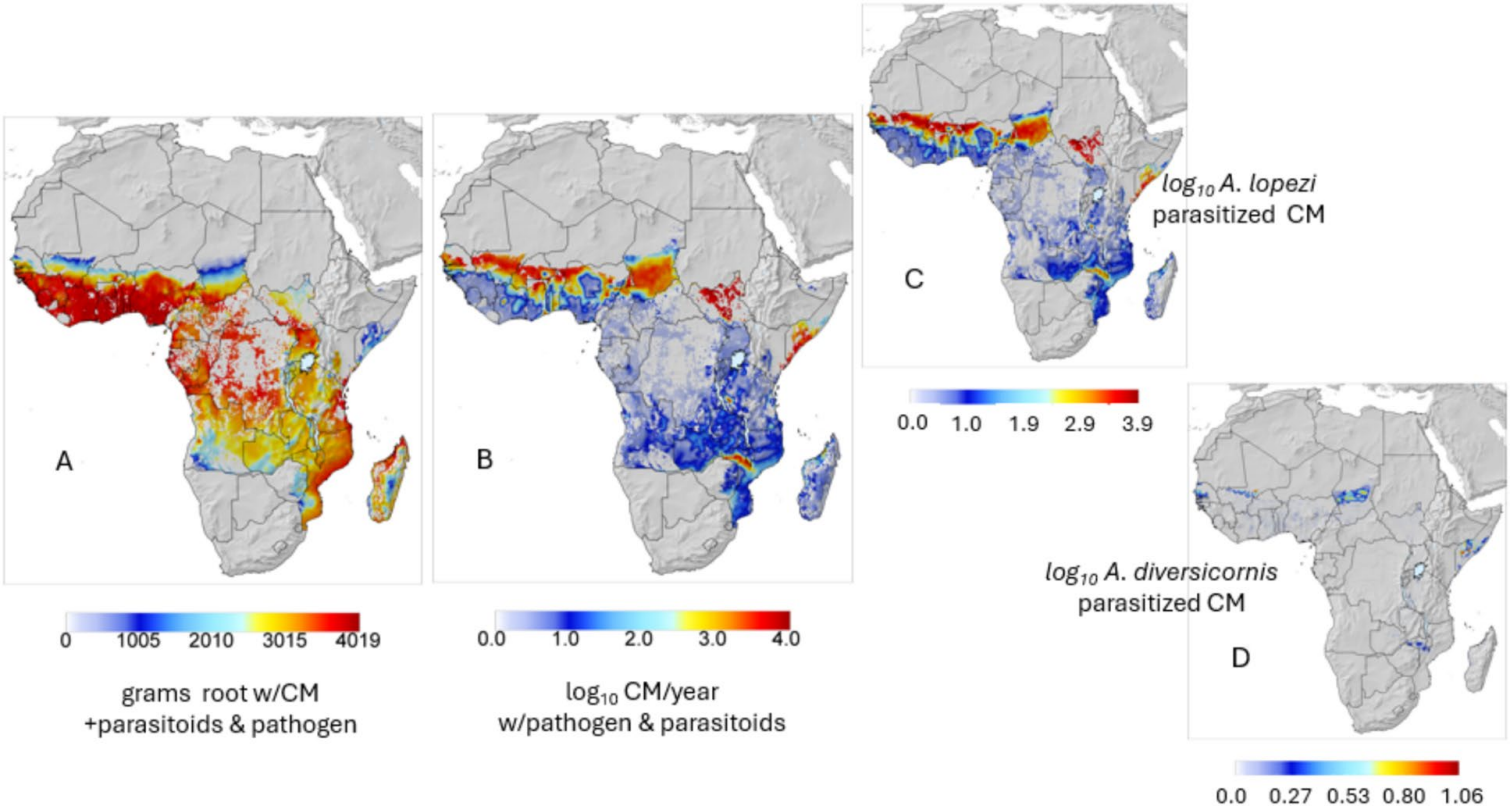
Prospective annual averages per plant during 1981–1990 after the release of *A. lopezi* and *A. diversicornis*: (**A**) relative root yield (g dry matter), (**B**) log_10_ cumulative CM active stages per year, (**C**) log_10_ cumulative *A. lopezi* parasitized mealybugs, and (**D**) log_10_ cumulative *A. diversicornis* parasitized mealybugs. The effect of the endemic fungal pathogen on CM is included in all sub figures, and all results are masked for the distribution of cassava^35^.

Prospective yields across the cassava belt rebounded to ~ 95% of pre-CM invasion levels (Figs. 3A vs. 5A) due principally to the control of CM by *A. lopezi* (Figs. 3B vs. 5B). The distribution and log_10_ average cumulative densities of *A. lopezi* and *A. diversicornis* post control of CM are mapped in Figs. 5C,D respectively showing the distribution of *A. lopezi* is wider and its densities considerably higher than that of *A. diversicornis*. High CM densities are predicted in drier northern and eastern areas where cassava production is marginal due to lower rainfall that reduces parasitoid and pathogen efficacy (see discussion).

*Marginal analysis of cassava yield with CM*—Data for lattice cells with average root yield greater than 1500 g per plant (i.e., hereafter called the cassava belt) are used here and in all later analyses. The dependent variable *Y* in Eq. 1 is grams root dry matter per plant while the independent variables ($x_{{_{i} }}^{ + } = [0, \, 1]$) indicate absence or presence values for the species associated with specific *Y* values. The independent variables ($x_{{_{i} }}^{ + }$) are mealybug (*CM*^+^), *A. lopezi* (*Al*^+^), *A. diversicornis* (*Ad*^+^), and pathogen (*P*^+^). Similar notation is used in all later analyses (see methods)

1 $$\begin{gathered} grams \, root = 3464.8 - 1085CM^{ + } + 958.0Al^{ + } + 666.1Ad^{ + } + 378.2P^{ + } \hfill \\ \quad \quad \quad \quad \quad - 687.9Ad^{ + } Al^{ + } - 260.5Ad^{ + } P^{ + } - 317.7Al^{ + } P^{ + } + 220.9Ad^{ + } Al^{ + } P^{ + } \hfill \\ \quad \quad \quad \quad \quad R^{2} = 0.171, \, F = 9,023.1, \, df = 350,442 \hfill \\ mean \, (t \, value): \, \hfill \\ CM^{ + } = \, 0.860 \, ( - 210.1), \, Al^{ + } = { 0}{\text{.533 }}(182.1), \, Ad^{ + } = \, 0.386 \, (25.3), \hfill \\ P^{ + } = \, 0.499 \, (68.7), \, Ad^{ + } Al^{ + } = \, 0.266 \, ( - 26.1), \, Ad^{ + } P^{ + } = 0.253( - 9.6), \, Al^{ + } P^{ + } = 0.268( - 43.3), \hfill \\ Ad^{ + } Al^{ + } P^{ + } = 0.132 \, (8.1), \hfill \\ \end{gathered}$$

Using average values for the $$x_{{_{i} }}^{ + }$$, the marginal average root dry matter loss per plant due to CM is $${\raise0.5ex\hbox{$\scriptstyle {\partial Y}$} \kern-0.1em/\kern-0.15em \lower0.25ex\hbox{$\scriptstyle {\partial CM^{ + } }$}}$$ = − 1085 g, while the average marginal contributions to yield recovery by *Al*^+^, *Ad*^+^ and *P*^+^ are $${\raise0.5ex\hbox{$\scriptstyle {\partial Y}$} \kern-0.1em/\kern-0.15em \lower0.25ex\hbox{$\scriptstyle {\partial Al^{ + } }$}}$$ = 575.8 g, $${\raise0.5ex\hbox{$\scriptstyle {\partial Y}$} \kern-0.1em/\kern-0.15em \lower0.25ex\hbox{$\scriptstyle {\partial Ad^{ + } }$}}$$ = 233.1 g, and $${\raise0.5ex\hbox{$\scriptstyle {\partial Y}$} \kern-0.1em/\kern-0.15em \lower0.25ex\hbox{$\scriptstyle {\partial P^{ + } }$}}$$ = 153.8 g respectively for a total of 962.7 g. The positive contribution of *A. diversicornis* to yield occurs because *Al*^+^ was absent in about half of the simulations, and *A. diversicornis* partially suppresses CM (e.g., Fig. 4B). Given *Al*^+^, the expected contribution of *Ad*^+^ is low (Fig. 5D) as seen by excluding *Ad*^+^ from the analysis in Eq. 2.

2 $$\begin{gathered} grams \, root = 3465.0 - 1091.0CM^{ + } + 955.0Al^{ + } + 596.7P^{ + } - 557.3Al^{ + } P^{ + } \hfill \\ \quad \quad \quad \quad \quad R^{2} = 0.155, \, F = 16,126, \, df = 231,295 \hfill \\ mean \, (t \, value): \, \hfill \\ \quad \quad \quad \quad \quad CM^{ + } = 0.860 \, ( - 209.39), \, Al^{ + } = {0}{\text{.533 }}(202.77), \, P^{ + } = \, 0.499 \, (123.9), \, Al^{ + } P^{ + } = 0.268( - 94.0) \hfill \\ \end{gathered}$$

Using average values,$${\raise0.5ex\hbox{$\scriptstyle {\partial Y}$} \kern-0.1em/\kern-0.15em \lower0.25ex\hbox{$\scriptstyle {\partial Al^{ + } }$}}$$ = 676.9 g and $${\raise0.5ex\hbox{$\scriptstyle {\partial Y}$} \kern-0.1em/\kern-0.15em \lower0.25ex\hbox{$\scriptstyle {\partial P^{ + } }$}}$$ = 299.7 g for a combined marginal compensation to yield of 976.0 g, which is roughly the same as in Eq. 1. The contribution of $$Al^{ + }$$ to yield is ~ 2.25-fold that of $$P^{ + }$$ supporting field observations^28,29,39,40^. The positive effect of $$P^{ + }$$ are due to rainfall increasing both plant growth and fungal mortality to CM.

*Control of CM*—Cumulative daily CM per plant per year (i.e., CM) is the dependent variable, and $Al^{ + }$, $P^{ + }$ are the independent variables in Eq. 3.

3 $$\begin{gathered} CM = {0}{\mathrm{.36616}}x10^{7} - 0.3370x10^{7} Al^{ + } + 0.3131x10^{6} P^{ + } - 0.4914x10^{6} Al^{ + } P^{ + } \hfill \\ \quad \quad \quad \quad \quad R^{2} = 0.119, \, F = {34,083}{\mathrm{.1}}, \, df = 760,116 \hfill \\ mean \, (t \, value): \, \hfill \\ \quad \quad \quad \quad \quad Al^{ + } = \, 0.518 \, ( - 211.0), \, P^{ + } = \, 0.495 \, (19.0), \, Al^{ + } P^{ + } = 0.259 \, (21.4) \hfill \\ \end{gathered}$$

Using mean values, $${\raise0.5ex\hbox{$\scriptstyle {\partial CM}$} \kern-0.1em/\kern-0.15em \lower0.25ex\hbox{$\scriptstyle {\partial Al^{ + } }$}} = - 3,613,246CM$$ and $${\raise0.5ex\hbox{$\scriptstyle {\partial CM}$} \kern-0.1em/\kern-0.15em \lower0.25ex\hbox{$\scriptstyle {\partial P^{ + } }$}} = 58,555CM$$ showing the large suppressive effect of *Al*^+^ on CM, while the positive effect of *P*^+^ is the net effect of precipitation on pathogen mortality to CM and the enhancement of plant growth that increases CM. The combined action of *Al*^+^ and *P*^+^ reduces average cumulative CM active stages ~ 97%.

### Biological control of cassava green mite

**Fig. 6:**
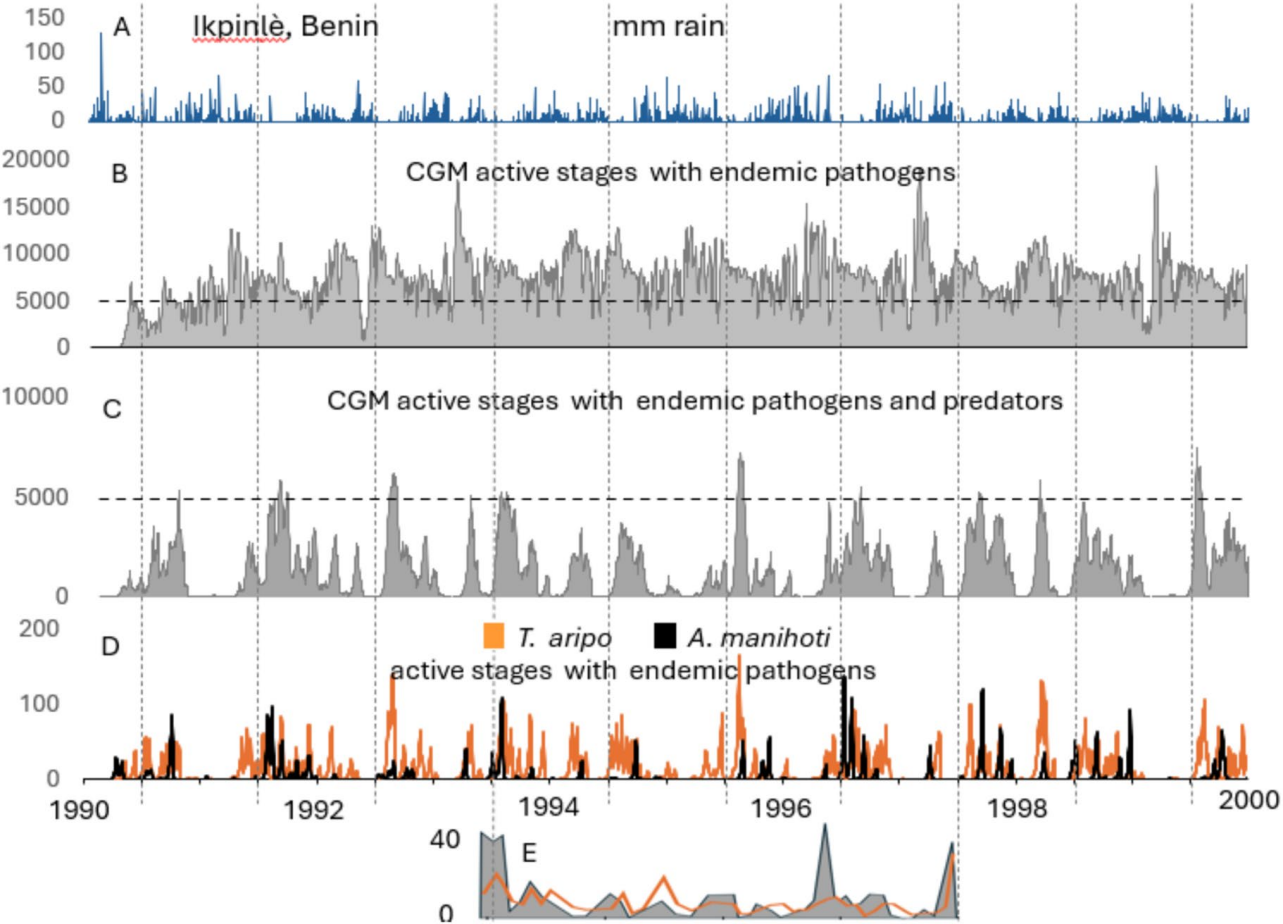
Simulated population dynamics of CGM and introduced predators per plant given the combined action of rainfall and endemic fungal pathogens using weather data for Ikpinlè, Benin during 6/1/1990 to 6/30/2000: (**A**) mm of daily rainfall, (**B**) CGM active stages absent predation, and (**C**) CGM with *T. aripo* and *A. manihoti*, (**D**) dynamics of *T. aripo* and *A. manihoti* active stages, and (**E**) field observations on 30 plants at approximately monthly intervals of average active stage counts of CGM (grey area) on the first developed leaf and *T. aripo* (orange line) in the shoot tips (see Supplemental Material for CGM and predator within plant distribution and behavior). The linear regression of data in Fig. 6E is $T. \, aripo = 0.457CGM,{\text{ R}}^{2} = 0.675$ (Yaninek, unpublished data). The horizontal lines at 5000 in 6B and 6C are reference lines.

The simulated dynamics of the cassava/CGM/predator system at the initial release site of Ikpinlè, Benin during 6/1/1990–6/30/2000 are summarized in Fig. 6. Before the introduction of the exotic predators, CGM mortality was low and due largely to the interaction of rainfall and an endemic fungal pathogen^41,42^ (Fig. 6A and B). After the introduction of the mite predators, CGM densities greatly declined due to the action *T. aripo* and less to *A. manihoti*, with highest CGM densities occurring during the dry season (Fig. 6C; see^43^). The simulation results confirm field observations^31,44^ that *T. aripo* is the most effective predator (Fig. 6D). Monthly estimates of *CGM* and predator densities on thirty plants during 1994–1997 at Ikpinlè, Benin summarized in Fig. 6E shows the persistence of the *CGM*-*T. aripo* interaction over multiple seasons.

**Fig. 7:**
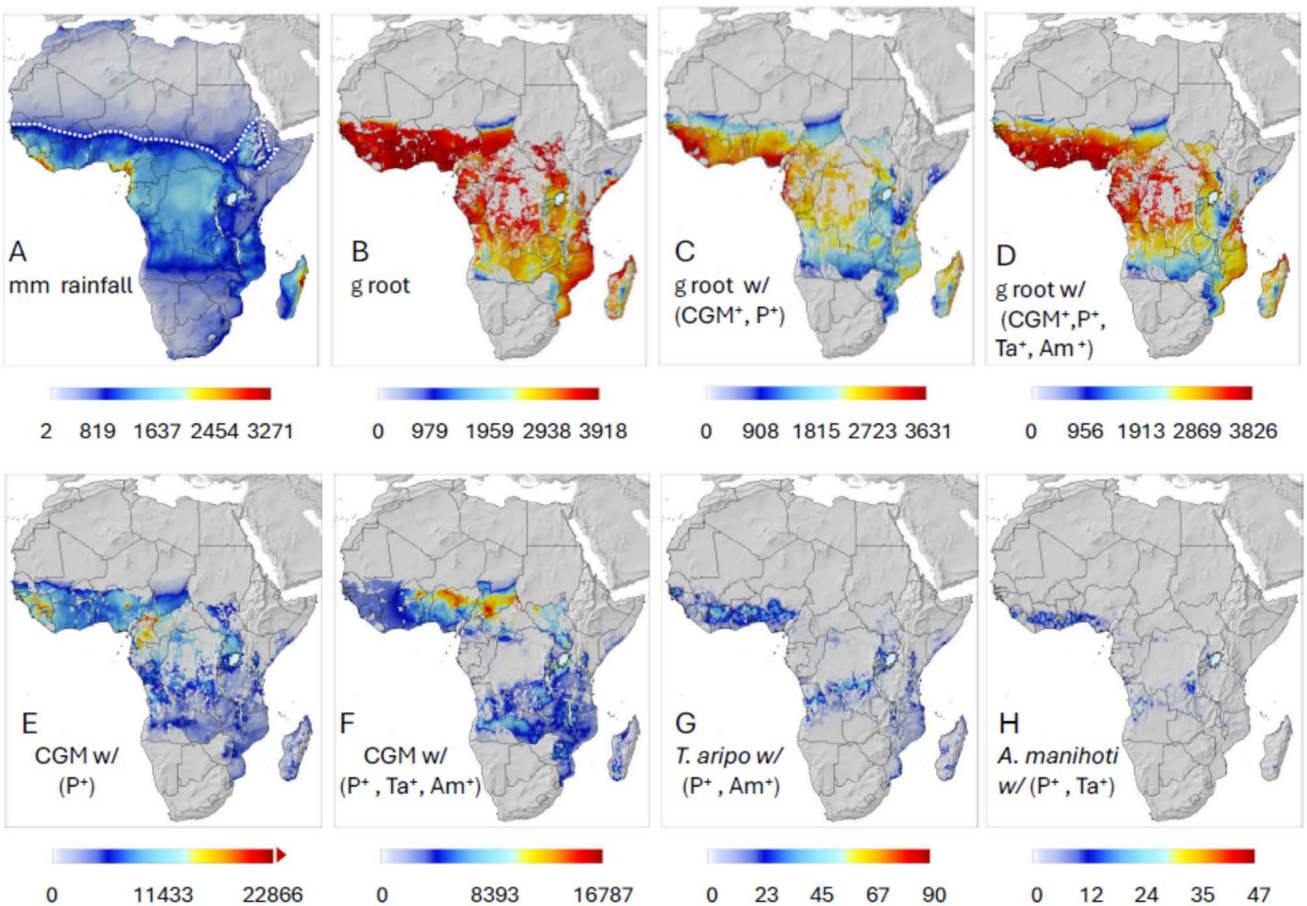
Simulated cassava system dynamics during 1991–2000: (**A**) average annual rainfall (mm) with the dashed line indicating the northern ~ 800 mm rainfall isohyet for the period, (**B**) prospective root yield (g dry matter per plant) absent CGM, (**C**) root yield with CGM^+^ and endemic pathogen *P*^+^, (**D**) root yield with CGM^+^, *P*^+^, *T. aripo* (*Ta*^+^), and *A. manihoti* (*Am*^+^), and (**E**) CGM active stages with *P*^+^, (**F**) CGM active stages with *P*^+^, *Ta*^+^ and *Am*^+^, (**G**) *T. aripo* active stages given *P*^+^ and *Am*^+^, and (**H**) *A. manihoti* active stages given *P*^+^ and *Ta*^+^. The simulation data are masked for the distribution of cassava^35^.

*Impact of cassava green mite*—Average annual rainfall during 1991–2000 is mapped in Fig. 7A, and prospective average cassava yields per plant absent CGM are mapped in Fig. 7B. Given CGM and the action of the endemic fungal pathogen, prospective cassava yields losses range from ~ 15% to ~ 67% (Fig. 7B vs. C) which agrees with measured yield losses of 13–80%^45^.

The introduction of the mite predators *T. aripo* and *A. manihoti* increased prospective root yields to ~ 95% of potential in many areas (Fig. 7B vs. D) due to control of CGM (Fig. 7E vs. F). Further, the prospective distribution of CGM (Fig. 7E) is comparable to that predicted by Parsa et al*.*^46^ using the species distribution MaxEnt algorithm^47^. The post introduction average densities of the two predators are summarized in Fig. 7G and H showing *T. aripo* has a wider distribution and its maximum densities are ~ 1.5–2.0 fold greater than *A. manihoti*.

*Marginal analysis of cassava yield with CGM*—The simulated cassava belt yield data is the dependent variable and absence-presence values for green mite (*CGM*^+^), *T. aripo* (*Ta*^+^), *A. manihoti* (*Am*^+^), and the pathogen (*P*^+^) are the independent variable in Eq. 4.

4 $$\begin{gathered} grams \, root = 3466.8 - 1345.0CGM^{ + } + 508.7Ta^{ + } + 289.0Am^{ + } + 488.8P^{ + } \hfill \\ \quad \quad \quad \quad \quad - 287.5Ta^{ + } Am^{ + } P^{ + } \hfill \\ \quad \quad \quad \quad \quad R^{2} = 0.224, \, F = 15674.5, \, df = 272,134 \hfill \\ mean \, (t \, value): \, \hfill \\ \quad \quad \quad \quad \quad CGM^{ + } = 0.816 \, ( - 266.2), \, Ta^{ + } = 0.449{ (}154.7),Am^{ + } = 0.439 \, (87.0) \hfill \\ \quad \quad \quad \quad \quad P^{ + } = 0.608 \, (133.2), \, Ta^{ + } Am^{ + } P^{ + } = 0.1581( - 58.8) \hfill \\ \end{gathered}$$

Substituting mean values in Eq. 4, average yield loss is ~ 23%, and the marginal effects are:$${\raise0.5ex\hbox{$\scriptstyle {\partial Y}$} \kern-0.1em/\kern-0.15em \lower0.25ex\hbox{$\scriptstyle {\partial CGM^{ + } }$}} = - 1345.0g$$, while $${\raise0.5ex\hbox{$\scriptstyle {\partial Y}$} \kern-0.1em/\kern-0.15em \lower0.25ex\hbox{$\scriptstyle {\partial P^{ + } }$}} = 432.1g$$, $${\raise0.5ex\hbox{$\scriptstyle {\partial Y}$} \kern-0.1em/\kern-0.15em \lower0.25ex\hbox{$\scriptstyle {\partial Ta^{ + } }$}} = 432.0g$$ and $${\raise0.5ex\hbox{$\scriptstyle {\partial Y}$} \kern-0.1em/\kern-0.15em \lower0.25ex\hbox{$\scriptstyle {\partial Am^{ + } }$}} = 210.5g$$ for a combined total of 1,074.6 g. This is an ~ 80% reduction in CGM damage (i.e., 1,074.6 g/1345 g). The large positive value for $${\raise0.5ex\hbox{$\scriptstyle {\partial Y}$} \kern-0.1em/\kern-0.15em \lower0.25ex\hbox{$\scriptstyle {\partial P^{ + } }$}}$$ is the net effect of rain increasing yield and pathogen mortality on CGM. The negative interaction term *Ta*^+^*Am*^+^*P*^+^ is reflective of interspecific competition resulting in a ~ 1.3% (i.e., 45.5 g) reduction in average yield.

*Control of CGM*—Using annual cumulative CGM eggs plus immature stages per plant as the dependent variable and *Ta*^+^*, Am*^+^, *P*^+^ as independent variables gives Eq. 5.

5 $$\begin{gathered} CGM = 80,381.0 \, - 0.1042 \times 10^{6} Ta^{ + } - 0.5160 \times 10^{5} Am^{ + } + 0.1791 \times 10^{6} P^{ + } \hfill \\ \quad \quad \quad \quad \quad + 0.3105 \times 10^{6} Ta^{ + } Am^{ + } - 0.2618 \times 10^{6} Ta^{ + } Am^{ + } P^{ + } \hfill \\ \quad \quad \quad \quad \quad R^{2} = 0.303, \, F = 23,659.0, \, df = 272,135 \hfill \\ mean \, (t \, value): \, \hfill \\ \quad \quad \quad \quad \quad Ta^{ + } = 0.450 \, ( - 154.4), \, Am^{ + } = 0.439 \, ( - 75.6), \, P^{ + } = 0.608 \, (293.3), \hfill \\ \quad \quad \quad \quad \quad Ta^{ + } Am^{ + } = 0.291 \, (232.4), \, Ta^{ + } Am^{ + } P^{ + } = 0.158 \, ( - 283.8) \hfill \\ \end{gathered}$$

The coefficients of *Ta*^+^*, Am*^+^ and *Ta*^+^*Am*^+^*P*^+^ are negative indicating they reduce CGM levels, while the interaction term *Ta*^+^*Am*^+^ is positive and is reflective of intraguild predation or competition. The coefficient for *P*^+^ is positive due to the net effect of rainfall favoring plant growth and the impact of pathogen mortality on *CGM*. Substituting average values in Eq. 5, the average annual cumulative CGM stages is 168,412 CGM per plant. The marginal effects of *Ta*^+^*, Am*^+^*, P*^+^ are $${\raise0.5ex\hbox{$\scriptstyle {\partial CGM}$} \kern-0.1em/\kern-0.15em \lower0.25ex\hbox{$\scriptstyle {\partial Ta^{ + } }$}} = - 37,768 \, CGM$$, $${\raise0.5ex\hbox{$\scriptstyle {\partial CGM}$} \kern-0.1em/\kern-0.15em \lower0.25ex\hbox{$\scriptstyle {\partial Am^{ + } }$}} = \, 16,496 \, CGM$$, $${\raise0.5ex\hbox{$\scriptstyle {\partial CGM}$} \kern-0.1em/\kern-0.15em \lower0.25ex\hbox{$\scriptstyle {\partial P^{ + } }$}} = \, 102,916 \, CGM$$ respectively.

Removing *P*^+^ to assess the action of the two predators alone yields Eq. 6.

6 $$\begin{gathered} CGM = 285,830 \, - 0.1274 \times 10^{6} Ta^{ + } - 0.7035 \times 10^{5} Am^{ + } + 0.6769 \times 10^{5} Ta^{ + } Am^{ + } \hfill \\ \quad \quad \quad \quad \quad R^{2} = 0.293, \, F = 16,459, \, df = 119,011 \hfill \\ mean \, (t \, value): \, \hfill \\ \quad \quad \quad \quad \quad Ta^{ + } = 0.576 \, ( - 191.7), \, Am^{ + } = 0.524 \, ( - 101.4), \, Ta^{ + } Am^{ + } = 0.288 \, (74.3) \hfill \\ \end{gathered}$$

The marginal impact of *Ta*^+^ ($${\raise0.5ex\hbox{$\scriptstyle {\partial CGM}$} \kern-0.1em/\kern-0.15em \lower0.25ex\hbox{$\scriptstyle {\partial Ta^{ + } }$}} = - 91,930.4 \, CGM$$) is ~ threefold that of *Am*^+^$$({\raise0.5ex\hbox{$\scriptstyle {\partial CGM}$} \kern-0.1em/\kern-0.15em \lower0.25ex\hbox{$\scriptstyle {\partial Am^{ + } }$}} = - 31,360.5 \, CGM)$$ and combined both predators reduce prospective per plant CGM populations by ~ 43%. Substituting average values in Eq. 6, average annual cumulative CGM stages are 195,080 CGM vs. 168,412 CGM (Eq. 5), indicating an irreplaceable mortality from *P*^+^ of ~ 14%.

## Discussion

Annual losses worldwide from invasive species since 1960 are estimated at US$ 1.13 trillion^48^ with annual losses of more than US$ 140 billion in the USA^49^, and US$ 28 billion in the European Union, with projected losses of US$148 billion by 2040^50^. Loss of staple food crops (e.g., cassava, maize, millet, sorghum) and of veterinary and human health in Africa and other developing areas are large but not well documented^51,52^. Classical biological control of invasive species using introduced natural enemies^53^ is a common practice, and when successful, control is self-sustaining unless disrupted. An exemplar modern biological control success was of the highly destructive cassava mealybug (CM) and cassava green mite (CGM) in Africa^54^ that kept food insecurity at bay for more than 200 million people^40^. Depending on monetary scenarios, the economic gains from the control of CM alone ranged from US$ 10–30 billion, with benefit–cost ratios of ~ 200^55^ and ~ 370–740^56,57^ that continue to increase. In contrast, the cost of the biological control program for CM and CGM was ~ US$16 million or about eight US cents per person affected^20^.

But classical biological control programs have a high failure rate^2,3^ largely because natural enemies are released after host specificity screening without sufficient knowledge of their potential efficacy. Furthermore, analyses of natural enemy attributes in simple models fail to inform efficacy in specific cases^5,7^. A major contributing factor in the failures is that target invasive species may have wide native ranges with numerous strains adapted to local conditions^58^, with natural enemies adapted to them. Because the initial invasion of a novel area may come from any part of the range, finding the most efficacious strains of natural enemies often proves vexing. A documented example is the walnut aphid (*Chromaphis juglandicola* Kaltenbach) where the temperature dependent vital rates of the parasitoid *Trioxys pallidus* (Haliday, 1833) introduced from France were ill suited to the hot environmental conditions of the Central Valley of California, while an Iranian strain provided excellent control^59^. Physiological incompatibility of a parasitoid to its host may also occur as found in the alfalfa weevil (*Hypera postica* (Gyllenhal))^60^.

The biology matters, and including the relevant complexity in weather-driven models is not difficult. By way of illustration, we show that an *ex-ante* analysis using our cassava system model and weather data from the invaded area would have given the correct predictions concerning the efficacy of the natural enemies for both CM and CGM across Africa. Specifically, the analyses capture astonishingly well the observed distribution and relative abundance of cassava, and of CM and CGM and their natural enemies, and their effects on cassava yield across Africa; all independent of species distribution records. The model captured the role of the biology, behavior, and interactions of the parasitoids *A. lopezi* and *A. diversicornis* in the control of CM (see^21,26^) and showed that natural enemies, rather than weather or resistant varieties, caused the decline of CM populations. The analysis explains why *A. lopezi* introduced from a small region in the Rio de la Plata Valley in South America quickly established with immediate high impact on CM across the ecological zones of Africa^27,57^. However, the analysis also shows that if only *A. diversicornis* had been introduced, partial control of CM would have resulted in failure (see Figs. 4 and 5).

The search for predators of CGM was focused in Brazil using a climate matching program developed at CGIAR/CIAT in Colombia^61^. Though eleven species of mite predator were introduced to Africa, only three species became established, with *A. manihoti* and *T. aripo* spreading widely and *T. aripo* becoming the dominant predator in the field^62^. This occurred despite *A. manihoti* having higher demographic parameters. The analysis explains that the capacity of *T. aripo* to use plant resources (e.g., pollen and likely exudates) as an alternative food source was responsible for its success (see Supplemental Materials). Hence, if only *A. manihoti* had been introduced, only partial control of CGM would have occurred, resulting in failure. Furthermore, the model predicts, as observed^28^, that populations of CM (Fig. 5B) and CGM (Fig. 7F) may still develop in areas with marginal rainfall as mortality from fungal pathogens decrease.

Complex food webs abound in agroecological and natural systems with myriad interactions across trophic levels^63^, and the entry of an invasive species at any trophic level may disrupt trophic interactions and seemingly present complexity beyond the capacity of PBDM analysis. But systems like alfalfa (*Medicago sativa* L.) have numerous food webs, and when the invasive spotted alfalfa aphid (SAA, *Therioaphis trifolii* (Monell)) invaded California, it severely disrupted the system regionally. A PBDM analysis of the natural enemies introduced for control of SAA enabled parsing the interacting but differing contributions of three parasitoids, a fungal pathogen and native coccinellid predators to the control of SAA across the different ecological zones where alfalfa grows. With control, the system recovered^10^, but this might not be the case in systems where critical components of food webs are irreparably changed or destroyed.

Well-documented PBDM analyses of natural enemy efficacy include the coffee (*Coffea arabica* L.) system. The analysis explained why several introduced parasitoids of coffee berry borer (*Hypothenemus hampei* (Ferrari)) in the Americas fail to control the pest, but also enabled exploration of alternative effective harvest and cleanup control strategies^64^. Using a simple model, Abram et al.^65^ posited that egg parasitoids of the invasive Asian brown marmorated stink bug (*Halyomorpha halys* Stål) were unlikely to control it, while a tri-trophic PBDM system explained why, and further posited *ex-ante* that despite low levels of parasitism, tachinid parasitoids of late nymphal and of adult stages would augment egg parasitism and have an important role in suppressing the pest across its vast Palearctic range^66^. Similarly, only partial control of yellow starthistle (YST; *Centaurea solstitialis* L.) in North America occurred despite extensive releases of several seed head insects that insufficiently reduced seed survival. An *ex-ante* PBDM analysis of the YST system posits that control would be enhanced by the introduction of the rosette weevil (*Ceratapion basicorne* (Illiger)) that kills whole plants and further reduces seed production in survivors^67^. These examples and others summarized in the Supplemental Materials in Gutierrez et al.^15^ strongly suggest that *ex-ante* in silico analyses of natural enemy efficacy with well parameterized mechanistic models such as PBDMs using weather data from invaded regions would be strategic and cost-effective and fill a critical need enhancing global food security.

Nevertheless, the physiological and behavioral parameters of the interacting species determine success or failure, and they vary widely. Hence, the biology matters and capturing it for each species in a food chain or web is crucial in predicting efficacy—no shortcut rules are apparent, but general methods for assembling PBDMs are increasingly available^15^. *Ex-ante* analyses of natural enemies in their native environments would further streamline field-based pre-release risk assessments^68,69^, with species distribution^70^ and genomic analyses^71^ being complementary. Such holistic analyses would further lower risk in natural enemy introduction^4^.

While PBDM development is perceived as difficult^72^, the math is simple, and the process is quite straightforward^15,73^. The bottleneck is often finding appropriate data in the literature to parameterize PBDMs and/or the inability to develop the biological data in house—especially in the pest’s native environments. Further, studies on species biology are often deemed mere technical work and are not fully appreciated or rewarded professionally, and yet such data are key to answering larger ecological/economic/social questions (see^74^). To facilitate progress in PBDM development, Python-based software is being developed to enable non-experts to develop PBDMs^75^, and appropriate weather files to drive the models are increasingly available. Textbooks outlining the mathematics are available^14,76^.

Among the myriad pest problems requiring holistic agro-eco-social analyses^76^ is whitefly (*Bemisia tabaci* (Glenn.)) that vectors mosaic disease and brown streak disease that currently constrain cassava production in Africa. Control of *B. tabaci* in resource-poor African cassava requires control methods that are low-cost and effective, such as traditional host-plant resistance and biological control—methods that are sustainable and readily disseminated^77^. The PBDMs for cassava and for the complicated biology of whitefly parasitoids^78^ could be used as a starting point to deconstruct this problem.

As epilogue, the PBDM approach tends toward reductionism, and from a theoretical standpoint has roots in thermodynamics with an explicative basis for population interactions provided by bioeconomic supply–demand principles common to all economies including those of humans^79–81^. This framework may provide a mechanistic pathway for *ex-ante* assessments of a natural enemy efficacy that can dramatically increase the return on investment in biological control and empower its use as an important tool in sustainable, climate-resilient agriculture; to move classical biological control beyond 'release-and-hope for the best stage’—to increase ecological stability in agroecological and natural systems with greater foresight and precision. This was the *raison d’être* of our study, and while we caution that no model can be a one to one description of nature, we recognize that—as stated by Heisenberg^82, cited from 83^—"what we observe is not nature itself, but nature exposed to our methods of questioning".

## Methods

PBDMs are time-varying life tables^10,12,80^ that capture the weather-driven daily development and temporal dynamics of species independent of time and place, a property that enables their use in climate change and GIS contexts. Weather-driven PBDMs of the cassava subsystems (Fig. 1A) linked by dry matter and energy flow to age-structured populations of plant subunits (Fig. 1B) were used in our study (^80^, see^84^). Cock et al.^85^ (see^86^) report a simulation model that predicts cassava growth and dry matter allocation to plant subunits but lacks demographic structure, limiting linkages to higher trophic levels. The cassava system model used is a meta-population composed of a population of plants (100 maximum, Fig. 1C) with each potentially having the full complement of interacting arthropod populations^16^ in the thousands of lattice cell across vast areas and ecological zones of Africa. As implemented, the PBDMs capture the weather driven, time varying bio-economics (supply/demand relationships) of species resource acquisition and allocation^79,81^.

**Fig. 8:**
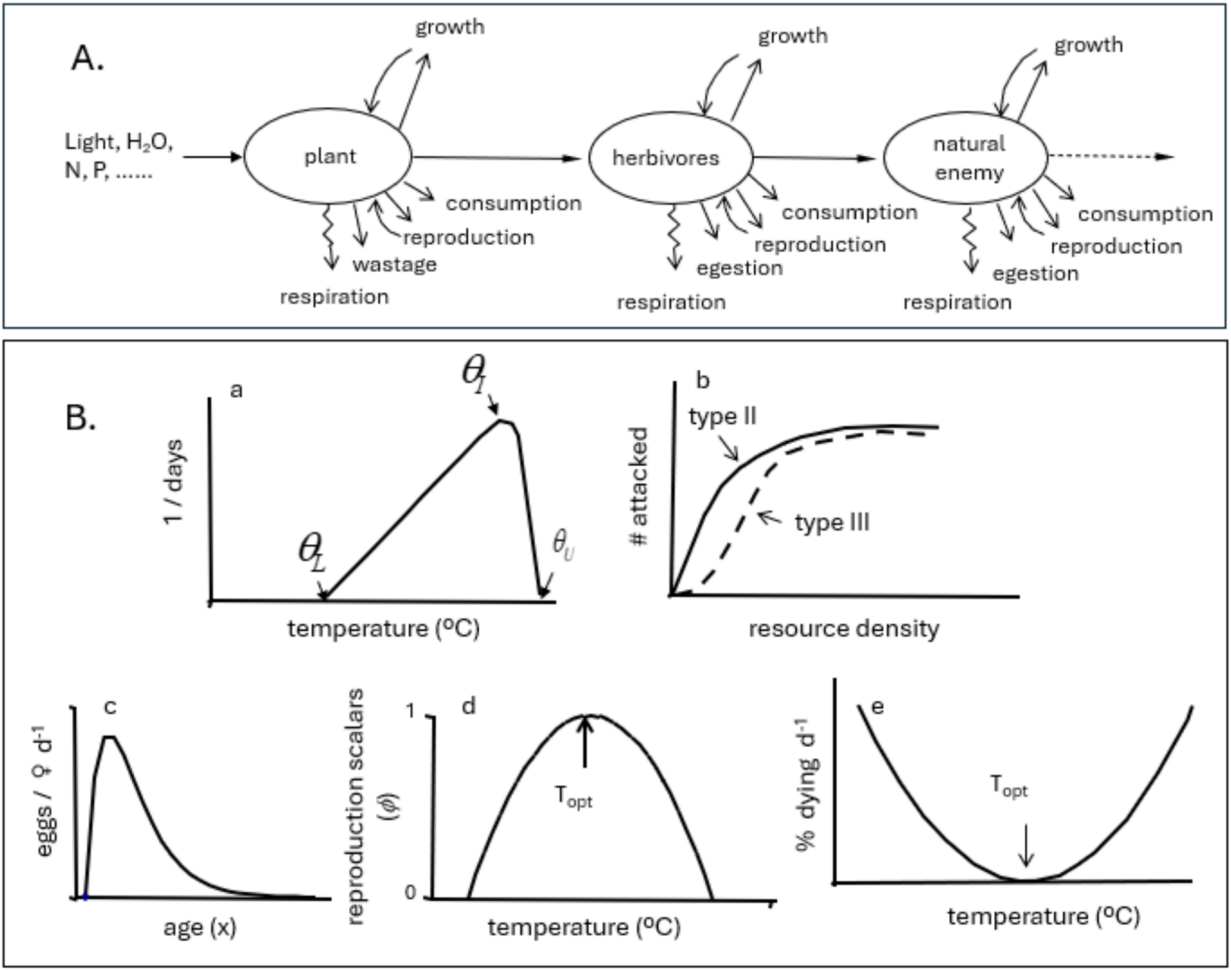
PBDM submodels: (**A**) schema for bio-economics of energy flow in a linear tri-trophic system (see text for allocation priorities), and (**B**) stylized biodemographic functions (BDFs) used to develop PBDMs: [(Ba) is the rate of development on temperature, (Bb) is the functional response for resource acquisition, (Bc) the age-specific reproductive profile at the optimum temperature, (Bd) the temperature scalar to correct reproduction from the optimum temperature, and (Be) is the mortality rate per day on temperature]. Note that similar shaped scalars to (Bd) and (Be) can be developed for the effects of relative humidity and other factors that in practice are not symmetrical.

Cassava variety IITA TMS30572 is used as a generic prototype with movement of arthropods between plants modeled as a function of species-specific resource supply–demand ratios on each plant. The only change to our model^16^ is the use of a nonlinear developmental rate data for cassava^36^ that captures the limiting effects of high temperature. The cassava system model is modular, and any combination of species with links to cassava and as appropriate to each other can be introduced in the computations using Boolean variables. The system model was coded in Borland Delphi Pascal 3. The two major components of PBDMs are: the age/mass structured dynamics model and the parameterized biological functions capturing the biology of each species.

*Population dynamics model*—The dynamics model accommodates age-mass structure and temperature-dependent developmental rates, and considerable aspects of the biology and behavior. Cohorts of ectotherm organisms have distributed maturation times with means and variance, and each stage/species may have different responses to temperature, resulting in different time scales for each. To accommodate the developmental biology and the behavior and physiology of the species, we use the time-invariant distributed maturation time dynamics model with attrition^87^ (see Supplemental materials), but the time-varying form is also available^88^. Other dynamics models may also be used^12,89,90^. Two approaches are used to parameterize the dynamics models: the metabolic pool (MP, Fig. 8A) and the biodemographic function approach (BDF, Fig. 8B)^15^.

PBDM/MP models capture per capita biomass/energy acquisition and allocation to wastage, respiration, growth and reserves if immature and to reproduction in adults (Fig. 8A), and the effects of mortality on plant and arthropod populations. Plants search for light, water, and nutrients to produce photosynthate that is allocated to plant subunit vegetative growth and reproduction^91^. The PBDM/MP approach was first used to develop a cotton canopy model composed of linked age-mass structured populations of leaves, stem, root, and fruit^92*,*93^, and later to develop models of the growth, development, and reproduction of pest arthropods^11^.

In PBDM/BDF models, the rates of development, births, and deaths of the species are estimated from age-specific life table studies conducted under an array of temperatures and other conditions^73^, but field studies can also be used^94^. The vital rates are the outcomes over the life cycle of a cohort of organisms of how they acquire and allocate energy, survive, and reproduce under the experimental conditions—i.e., the results of metabolic pool processes under the experimental conditions. BDFs summarize the effects of abiotic variables on species developmental, birth, and death rates across environmental conditions, and are used to parameterize the dynamics models. Common BDFs are depicted in a stylized manner in Fig. 8B, but other BDFs may be developed to accommodate additional aspects of the biology of species (e.g., the response to relative humidity; see Supplemental Materials). Temperature-dependent development (Fig. 8Ba) is used in both MP and BDF approaches to estimate daily increments of species-specific physiological time and age. The migration rates of arthropods between plants in both MP and BDF approaches are functions of species-specific resource acquisition success (i.e., 0 < supply/demand < 1).

These methods were used to model our cassava system, noting that most of the species models are MP-based.

*Weather data*—The system model is run using daily maximum and minimum temperature, precipitation, solar radiation, and relative humidity as inputs (see Supplemental Materials). Daily weather data for 40,691 ~ 25 × 25 km georeferenced lattice cells across Africa for the 1980–2010 period were sourced from the open access AgMERRA global weather dataset created by the Agricultural Model Intercomparison and Improvement Project (AgMIP, https://agmip.org/)^95^. The AgMERRA data were accessed through the Goddard Institute for Space Studies (GISS) of the National Aeronautics and Space Administration (NASA, https://data.giss.nasa.gov/impacts/agmipcf/). From the perspective of ectotherms, climate change is simply another weather pattern, but the effects of climate change on the system are beyond the scope of this study (see examples^15^).

*Simulations—*The time step in the model is a day in physiological time units that differ daily for each species. The computation time for the daily dynamics of a 100-plant system per year at one location is ~ 6–10 s on a laptop computer, making the total time over an eleven-year period across 40,691 lattice cells inordinately long. Hence for feasible computations on a laptop computer, ten randomly spaced plants in 10,172 lattice cells in alternating latitude–longitude were used. This subset reduced the computation time considerably and yet provided sufficient grain to characterize the distribution and relative abundance of the species across Africa.

The biological control efforts against CM occurred during 1980–1990 and during 1990–2000 for CGM, and hence all combinations of the two subsystems were run separately for the two time periods. We note, however, all species can be run concurrently. The same nominal initial conditions for species were used for all lattice cells, and the species absence-presence values (i.e., [0, 1]) and selected annual georeferenced summary simulation variables for all populations were written to text files. Because the system was assumed equilibrating to lattice cell weather, data from the first year were excluded in computing lattice cell means, standard deviations, and coefficients of variation of the annual summary variables. Only means of summary variables are used in GIS mapping and the bioeconomic analyses.

*GIS mapping*—The open source GIS software GRASS^96,97^ (http://grass.osgeo.org/) was used to map the summary output data. The map of the current distribution of cassava cultivation in Africa^35^ is used in our analyses for comparative purposes. Other GIS data mapping layers were sourced from the public domain repository *Natural Earth* (https://www.naturalearthdata.com/). Inverse distance weighting bicubic spline interpolation was used to create a continuous raster surface of the simulation data.

*Bioeconomic analysis*—A general binomial multiple linear regression model (Eq. 7) was used to summarize the Africa-wide summary of annual simulation data. Root dry matter yield (grams) or pest density (CM, CGM) are the dependent variables (*Y*) and species absence-presence values are the *I* independent dummy variables (i.e., $x_{{_{i} }}^{ + }$, [0, 1]). Specifically,

7 $$Y = f(x_{1} , \, .....,x_{I} ) = a + \sum\limits_{i = 1}^{I} {b_{i} x_{{_{i} }}^{ + } } + \sum\limits_{j = 1}^{J} {c_{j} {}_{n}^{I} x_{j}^{ + } } + d\prod\limits_{i = 1}^{I} {x_{i}^{ + } } + U$$

where $${}_{n}^{I} x_{j}^{ + }$$ is the *j*th interaction of the $$x_{{_{i} }}^{ + }$$ taken *n* at a time, $$\prod\limits_{i = 1}^{I} {x_{{_{i} }}^{ + } }$$ is the product of the *I* variables, and *U* is the error term. Only significant regression terms (*p* ≤ 0.05) were included in the final model. The goal of the analyses was to estimate the marginal impact of species presence $$x_{{_{i} }}^{ + }$$ on cassava yield (g dry matter) and pest density estimated as $${\raise0.5ex\hbox{$\scriptstyle {\partial Yield}$} \kern-0.1em/\kern-0.15em \lower0.25ex\hbox{$\scriptstyle {\partial x_{i}^{ + } }$}}$$,$${\raise0.5ex\hbox{$\scriptstyle {\partial CM}$} \kern-0.1em/\kern-0.15em \lower0.25ex\hbox{$\scriptstyle {\partial x_{i}^{ + } }$}}$$ and $${\raise0.5ex\hbox{$\scriptstyle {\partial CGM}$} \kern-0.1em/\kern-0.15em \lower0.25ex\hbox{$\scriptstyle {\partial x_{i}^{ + } }$}}$$ given the average effects of the other independent variables.

## Supplementary Information

Supplementary Information.

## Data Availability

All the data used are available in the cited references, with the fitted BDF functions given in the supplemental materials. All the GRASS GIS geospatial data layers used in this analysis are available open access on Zenodo^34^ at 10.5281/zenodo.13254494.

## Code availability

The Borland Delphi Pascal 3 code for the cassava tri-trophic PBDM system is available open source on Zenodo^98^ at https://zenodo.org/records/17559583 (associated GitHub repository: https://github.com/casasglobal-org/pascal-pbdm-cassava). The code for the geospatial software based on GRASS GIS used for mapping and geospatial analysis is available open source on Zenodo^33^ at https://zenodo.org/records/17551910 (associated GitHub repository: https://github.com/casasglobal-org/casas-gis).
