# Supplementary material for: Predicting natural enemy efficacy in biological control using ex-ante analyses
Source: Sci Rep. 2025 Dec 29;15:44886. doi: 10.1038/s41598-025-29022-1 (PMC12748761; doi:10.1038/s41598-025-29022-1)
Supplement: Supplementary file 1 — Supplementary Information. [file 41598_2025_29022_MOESM1_ESM.pdf]

Supplemental Materials

Predicting natural enemy efficacy in biological control using ex-ante analyses

Andrew Paul Gutierrez, Luigi Ponti, Peter Neuenschwander, John S. Yaninek, Hans R. Herren

An important transformation in ecological research occurred when system thinking shifted the attention of ecologists from objects to relationships and from structures to processes - from objects to networks of relationships embedded in larger networks - and from fundamental structures affected by forces and mechanisms to the recognition that every structure is a manifestation of underlying processes[1].

Our analyses delve into the details of the biology of the cassava system, but the goal is not a one-to-one description of the biology of the system, rather the goal is to capture sufficiently the weather-driven relationships underpinning the system sufficiently to make prospective model predictions independent of time and place. Interested readers should review the cited papers for full details of the cassava system model.

Cassava mealybug and its natural enemies

The model is mostly based on laboratory studies [2–4], and describes the distribution and impact of the cassava mealybug (*Phenacoccus manihoti* Matile-Ferrero (Hemiptera, Pseudococcidae))(CM) and *Anagyrus lopezi* DeSantis (Hymenoptera, Encyrtidae) astonishingly well as reported in surveys across Africa [4–8]. About 10% of all fields develop damaging CM populations depending on the season and how long *A. lopezi* has been established, with outbreaks mostly confined to sandy, un-mulched soils. Mulching improves the condition of the plant and increases CM size and hence the performance of *A. lopezi* developing on them [9]. Indigenous (and introduced) predators as well as non-specific hyperparasitoids become locally abundant only under conditions of high mealybug infestation, but do not contribute measurably to CM control [7,10–14]. Predatory coccinellids removed about 10% of high peak CM populations [15] and hyperparasitoids behaved strictly in a density dependent manner, and hence they are mostly absent at the common low CM densities [11]. For this reason, they were not included in this study.

Laboratory studies indicate that *A. lopezi* out-competes *A. diversicornis* (Howard) in cases of multiple parasitism because it has a higher search efficiency, attacks smaller CM, and has a higher female-biased sex ratio under adverse conditions that produce smaller mealybugs [16]. These attributes enabled *A. lopezi* to dominate across Africa as shown by the model. The dominant role of *A. lopezi* was further documented in exclusion experiments [4,17,18] and reconfirmed when it was released in Southeast Asia for control of CM [19].

Cassava green mite and its natural enemies

As with CM, the model parameters for the cassava green mite (*Mononychellus tanajoa* (Bondar), (Trombidiformes, Tetranychidae)) (CGM) and its natural enemies are primarily from laboratory studies (see[20,21] for relevant citations) and the simulations reflect the known distributions and impact well. In its native range in the Neotropics, CGM populations are maintained under natural control by a combination of local predators, pathogens, and weather (e.g., rainfall mortality), especially where cassava has been locally selected and cultivated in a traditional manner [22–25].

After the accidental introduction of CGM in Africa, local natural enemies, largely opportunistic generalist predators like coccinellids in the genus *Stethorus* and staphylinids in the genus *Holobus* (= *Oligota*) [20] and endemic fungal pathogens never associated with epizootics [26] were present, but they were not effective biological control agents. Yield losses following the invasion ranged from 13 to 80% [27–31]. CGM feed on the under surface of young leaves killing leaf cells reducing the photosynthetically active leaf area with densities varying with plant drought stress, leaf age and cultivar [32,33,31]. Yaninek, Gutierrez *et al*. [32] found that at 24°C, average longievity of CGM adult female in Africa was ~ 32 days and produced ~58 eggs over ~16 days, while Moraes *et al.* [34] under similar conditions in the Neotropics found that at 24°C CGM adult female lived an average of ~ 30 days and produced ~42 eggs over ~15 days.

The interaction biology of CGM and its native predators is complicated. All the released mite predators (Mesostigmata, Phytoseiidae) that established in Africa were selected for introduction based on agrometeorological matching criteria [35]. Subsequent studies revealed that *Typhlodromalus aripo* De Leon, the apparently least effective predator because it was less voracious and slower in population increase than *Amblydromalus manihoti* Moraes and *Neoseiulus idaeus* Denmark & Muma, proved to be the best biological control agent for *M. tanajoa* in Africa.

Typhlodromalus aripo: *T. aripo* establishment, dispersal and persistence on cassava [36] is enhance by its efficient location of prey, and persists at low prey densities by feeding on pollen, and cassava extrafloral exudates as an alternative food sources [37]. It normally inhabits a refuge located in-between the leaf primordia at the apex of the cassava plant that is thought to provide protection (a refuge) against abiotic factors and intraguild predation [38]. *T. aripo* shows a pronounced diurnal within-plant change in distribution and elicit an avoidance response in mobile CGM stages causing them to disperse and becoming less abundant on the first twenty leaves below the apex during the night compared to the same leaves in late afternoon [36]. *T. aripo* also displays a marked attraction to odors emitted from either CGM-infested apices or infested young leaves compared to infested old leaves, but showed no preference for odors from apices versus young leaves [39]. Under high prey densities, the average longevity of *T. aripo*

adults is about twice that of *A. manihoti*, but as prey densities fall, reproduction in *T. aripo* declines but its longevity is double that of fully fed adults (see [40]). This occurs because *T. aripo* feeds on pollen and extrafloral exudates enabling it to extend its longevity when prey is scarce, but with reduced fecundity [41]. *T. aripo* exhibits a trade-off in its metabolic rate: it has a low metabolic rate which allows long survival periods but slows oviposition and development (*c.f.* [40]). This trade-off in biology is captured in the *T. aripo* model by scaling the daily change in physiological time (${}^{s}\Delta x(T(t))$, see below) and fecundity by the food supply/demand ratio (S/D) that decreases both the developmental rate and reduces fecundity. However, under abundant prey, the functional response of *T. aripo* females to CGM egg density is type II reaching an asymptote at ~175 CGM eggs attacked/day under experimental conditions [42]. However, its numerical response is quite low reaching a maximum of ~1.2 to 1.4 eggs/day at 100 CGM eggs attacked/day [42,43]. Interestingly, one *T aripo* egg is produced at 22 prey eggs attacked, and thereafter, the reproductive rate levels off at ~1.3 eggs/day despite a high attack rate. This suggests that at high prey densities, *T. aripo* can kill four-fold more prey eggs (or egg equivalents) than required for reproduction (see [42]). Further, Mutisya [44] found the thermal limits for *T. aripo,* are 11.4°C for development with oviposition occurring between 12°C and 33°C with the optimum at about 27°C. High fecundity occurs in the range 25 to 100% RH with an optimum at ~75% RH with an adult longevity of ~426 dd.

Amblydromalus manihoti: In sharp contrast, *A. manihoti* is found only on fully developed cassava leaves and preferentially forages in the middle of the foliage [45]. It is a generalist mite predator and does not discriminate between volatiles from different infested cassava leaves [39] lowering its search rate on cassava. It has a high metabolic rate; rapidly consumes prey that it converts to eggs at the expense of lower survival and longevity [43]. Furthermore, *A. manihoti* does not feed on pollen and readily migrates when prey populations on cassava are low [46]. The lower thermal threshold of *A. manihoti* is 6.64°C for development, and oviposition occurs between 6.64°C and 31°C with an optimum at about 19°C above 40% RH. The thermal parameters for *A. manihoti* are those often associated with a temperate species. Artificially increasing the lower thermal threshold to 11°C in the model did not measurably change the conclusions as the other aspects of the biology proved more important.

System model overview

Cassava model

The original cassava system model was a canopy model composed of age-mass structured population sub-models of leaf, stem, root (tuber), pests, and natural enemies/pathogens [47,48,20,15]. The canopy model was extended to be a meta-population model of up to 100 plants evenly or randomly spaced with each plant having different areas for growth and each potentially having the full complement of the arthropod species

in our study [21]. However, for daily computations over a ten-year period on a laptop computer, a system of ten randomly spaced plants was used in our study.

Plant models are usually constructed as metabolic pool models (MP) that capture the effects of per capita biomass/energy demand (D) and realized acquisition via search (S) and allocation to respiration, growth, and reproduction. Note that S<D. Specifically, plants search for light, water, nutrients to produce photosynthate that is allocated to plant subunit growth and to reproduction (*c.f.* [49]). Gutierrez and Baumgärtner [50] used MP notions to develop PBDM models for growth, development, and reproduction of arthropods. Most of the species in the cassava system model are MP based (Table 1).

Table 1. Type of model used for each stage/species (MP = metabolic pool; BDF = biodemographic functions).

**Species**	**PBDM model used**			
	**All stages**	**Eggs**	**Immatures**	**Adults**
Cassava	MP			
CM	MP			
A. lopezi		MP	MP	BDF
A. diversicornis		MP	MP	BDF
CGM	MP			
T. aripo	MP			
A. manihoti	MP			
Pathogens	BDF*			

*A function of daily rainfall.

Biodemographic functions (BDF) can also be developed to characterize developmental rates and birth and death rates of species (*c.f.* [51]). These vital rates are usually estimated from age-specific life table studies conducted under an array of temperatures and other conditions [52,53] and are the outcomes over the life cycle of a cohort of organisms of how they acquired and allocated energy, survived, and reproduced under the experimental conditions – i.e., metabolic pool processes. These functions can also be estimated from field ecological studies [54–58].

The BDFs commonly used to estimate the effects of abiotic variables on species developmental, birth and death rates in age-structured, weather-driven BDF models are depicted in a stylized manner in Fig. 8 in the text. Other BDFs can be developed to accommodate additional aspects of the biology of a species.

Developmental rate: Time and age for all species are in physiological time units that differ among the species. An improved component of the system model is a nonlinear developmental rate model for cassava where *r(T)* was estimated from developmental time data in Keating and Evenson [59].

$$r(T) = 1/d(T) = \frac{a(T - \theta_L)}{(1 + b^{T - \theta_I})} = \frac{0.0095(T - 14.85)}{(1 + 1.6^{T - 33.55})} \quad (1)$$

d(T) is development time in days at temperature *T*, with fitted constants a, b, $r(T)=0$ at $T \leq \theta_L$=14.85°C, the maximum developmental rate occurs at θ_I ~33.55°C, and θ_U= 41°C is the upper thermal threshold. Due to data limitations, linear developmental rate models (i.e., ${}^s r(T) = a + bT$) were used for the other species (left superscript s), with ${}^s r(T)=0$ at $T \leq \theta_L$. The physiological developmental time constant (${}^s\Delta$) in degree days (*dd*) for each life stage of a species was estimated in the linear range of favorable temperatures as ${}^s\Delta = d(T) \times (T - {}^s\theta_L)$ [60]. The daily increment of physiological time at time *t* and temperature *T* is ${}^s\Delta x(T(t)) = {}^s r(T(t)) {}^s\Delta$. Hence, on average a cohort of individuals initiated at time t_0 completes development when $\int_{t_0}^{t} {}^s r(T(t))dt = 1$ in continuous form, or $\sum_{t_0}^{t} {}^s\Delta x(T(t)) = {}^s\Delta$ in *dd* units in our discrete time models. Further, each species (and their stages) may age on different temperature-dependent time scales. As noted above, at low prey density, *T. aripo* increasingly feeds on pollen that increases longevity but decreases fecundity. This biology is incorporated in the model by using the success rate of *T. aripo* feeding on CGM prey (S/D<1) to scale ${}^{Ta}\Delta x(T(t))^{Ta}(S/D)$ (i.e., slowing the developmental rate) and population level fecundity (*E*) (see below).

Resource acquisition and allocation

All organisms are consumers. Stage-specific resource acquisition (S) is demand-driven (D) and may be in mass or number units. Population resource demands (D) for a species/stage (left superscript *s*) (Gutierrez [61]) are computed as

${}^s D = \sum_{i=2}^{7} {}^s N_i \, {}^s D_i \, {}^s \Delta x(T(t))$, where ${}^s D_i$ is the per capita demand dd^{-1} at age *i*, ${}^s N_i$ is the number of individuals of age *i*, and $\Delta x(T(t)$ is the *dd* at time *t* and temperature *T*. Population-level resource supply (S) obtained by species/stage (s) (text Fig. 8B) is computed using the predator form of the functional response model as

$${}^s S = {}^s D(1 - e^{\frac{-{}^s\alpha \, prey}{{}^s D}}) \quad \{2\}$$

where a is the consumer search rate.

Using the MP approach, the demand for a plant (${}^{plant}D$) or say a mealybug ${}^{CM}D$) in mass units would be the total of all age and temperature dependent subunit growth and reproduction demand ($D_G + D_R$) plus respiration costs (vM) corrected for assimilation efficiency ($(1-\beta)$).

$$ {}^{plant}D = (D_G + D_R + vM) / (1-\beta) \; where \; {}^{plant}S = {}^{plant}D(1 - e^{\frac{-\alpha \text{ resource}}{{}^{plant}D}}). \qquad \{3\} $$

This form assumes the resource attacked is not available to other consumers (e.g., light captured by leaves, prey consumed by a predator).

However, some resources may be attacked more than once. For example, a parasitoid female attacks whole individuals that may also be attacked more than once by the same or other parasitoids (super and multiple parasitism). In this case, ${}^{s}D$ is the per capita number of *hosts* an adult female parasitoid in each age class (i=1,…,7) can attack (i.e., its egg load), and ${}^{s}D = \sum_{i=2}^{7} {}^{s}N_i \, {}^{s}D_i \, {}^{s}\Delta x(T(t))$ is the population attack demand. Using the parasitoid form of the model where multiple attacks on the same host may occur,

$$ {}^{s}S = hosts \times proportion\ attacked = hosts(1 - e^{\frac{-{}^{s}D}{H}(1-e^{\frac{-{}^{s}\alpha H}{{}^{s}D}})}) \qquad \{4\} $$

where a is the parasitoid search rate and *H* is the available hosts. In all cases 0 < ${}^{s}(S/D)$ < 1.

In a more realistic model (eqn. 5), realized demand by a parasitoid population (${}^{s}D$**)** is the per capita fecundity (${}^{s}f(x)^{-dd}$) at age *x* at optimal temperature (T_{opt}) (see text Fig. 8C), ${}^{s}sr$ is the sex ratio and ${}^{s}\phi_T$ scales the demand for temperature effects in a time temperature varying environment.

$$ {}^{s}D = {}^{s}\phi_T \cdot {}^{s}sr \cdot \int {}^{s,adults}N \cdot {}^{s}f(x)^{-dd} dx \; {}^{s}\Delta x(T(t)) \qquad \{5\} $$

Variations of the above biology occur for plants and predacious organisms, but by analogy all of them can be accommodated by these simple functions [see 61].

Cassava mealybug life stages and parameters

The mealybug model is a mass-age structured MP model. Age windows for the CM stages in *dd*>14.6°C are:

0 114 182 306 371 890dd
|--eggs---|--crawlers--|--larv2, 3--|-- adults --|--adults ovip---|.

The total eggs produced per day by the CM population is:

$$ {}^{CM}E = {}^{CM}sr \, {}^{CM}\phi(T) \, {}^{CM}(S/D) \sum_{i=1}^{k_{CM}} ((7.1/(1+1.325^{i}))CM_i $$

where $0 \leq {}^{CM}\phi(T) = (T, \theta_L, \theta_U) = 1.0 - (T - \theta_L - A)/A)^2 \leq 1$ scales the effects of temperature on fecundity as a symmetrical scalar function of mean temperature T in the range $\theta_L = 18^{\circ}C$ to $\theta_U = 35^{\circ}C$, where A= (θ_L - θ_U)/2.0 (see text Fig. 8D), and ${}^{CM}(S/D)$ is the supply/demand ratio.

The notation ${}^{CM}\phi(T)$ is used for CM, but a similar notation with left superscript is used to denote the same functional form for the other species (e.g., ${}^{Al}\phi(T)$, ${}^{Ad}\phi(T)$, ${}^{CGM}\phi(T)$, etc.). Other similar scalar functions can be developed and their product used to scale fecundity (e.g., ${}^{CM}\phi(Nitrogen) \cdot {}^{CM}\phi(RH) \cdot {}^{CM}\phi(pH)$) or other biological processes.

Rainfall/fungal mortality on CM is a simple empirical model:

$$0 \leq {}^{CM}\mu_{P^+} = 0.45(1 - e^{-0.025\,precip}) \leq 1.$$

Cassava mealybug natural enemies

Prior studies (see above) indicated *A. lopezi* out competes *A. diversicornis* in cases of multiple parasitism, it has a higher search efficiency, attacks smaller CM, and has a higher female-biased sex ratio under adverse conditions that produce smaller mealybugs (see [15]). These attributes enabled *A. lopezi* to dominate across Africa.

***A. lopezi* life stages and parameters**

age := transit times for *A. lopezi* in *dd* >13.5°C

```
age1-------------age2--------age3------------------------------age4
0                100         186                               366dd
|--eggs ---larva-|--pupa----- | --------------------adults ---------|
```

$${}^{Al}D = {}^{Al}sr\,{}^{Al}\phi(T)\{{}^{Al}ovip(1 + {}^{Al}hf)\int {}^{Al}Adlt\;dx\}\,{}^{Al}\Delta x$$

where ${}^{Al}hf$ =0.2 corrects for host feeding, the symetrical concave function $0 \leq {}^{Al}\phi(T) = (T,\ 13.5,\ 34) \leq 1$ corrects for temperature limits of 13.5°C and 34°C, ${}^{Al}ovip$ = 0.9 is fecundity per *dd*, ${}^{Al}sr \geq 0.5$♀♀ is the sex ratio, and ${}^{Al}\Delta x$ is the change in age/time. Potential hosts (${}^{Al}H$) for *A. lopezi* weighted for *CM* stage preferences and multiple and super parasitism (preference for parasitoid egg-larval stages of *Al*[1] and *Ad*[1]) are computed as follows:

$${}^{Al}H = 0.328\,CM[3] + 0.514CM[4] + 1.000CM[5] + 1.000CM[6] + 0.25Al[1] + 0.25Ad[1]$$

where ${}^{Al}H$ replaces H in the parasitoid functional response eqn. 4.

$0 < {}^{Al}\mu = (1 - e^{\frac{-{}^{Al}D}{{}^{Al}H}(1 - e^{\frac{-{}^{Al}\alpha \; {}^{AL}H}{{}^{Al}D}})}) < 1$ and ${}^{Al}a = 0.65$ is the parasitoid search rate with ${}^{Al}Na = {}^{Al}H\,{}^{Al}\mu$ being the number of hosts attacked. The computations for attack rate ($0<{}^{s}\mu \leq 1$) of CM and parasitoid host stages attacked by *A. lopezi* corrected for preference follows:

$${}^{CM}\mu[3] = {}^{Al}Na \times (0.328\ CM[3]/{}^{Al}H)/CM[3]$$
$${}^{CM}\mu\,[4] = {}^{Al}Na \times (0.514\ CM[4]/{}^{Al}H)/CM[4]$$
$${}^{CM}\mu\,[5] = {}^{Al}Na \times (CM[5]\,/{}^{Al}H)/CM[5]$$
$${}^{CM}\mu\,[6] = {}^{Al}Na \times (CM[6]\,/{}^{Al}H)/CM[6]$$
$${}^{Al}\mu\,[1] = {}^{Al}Na \times (0.250\ Al[1]/{}^{Al}H)/Al[1]$$
$${}^{Ad}\mu\,[1] = {}^{Al}Na \times (0.250\ Ad[1]/{}^{Al}H)/Ad[1]$$

Similar computations occur for *A. diversicornis,* and if both parasitoids are present, the attacked hosts are distributed as illustrated by the Venn diagram below, noting that in cases of multiple parasitism, *A. lopezi* wins (i.e., the shaded area).

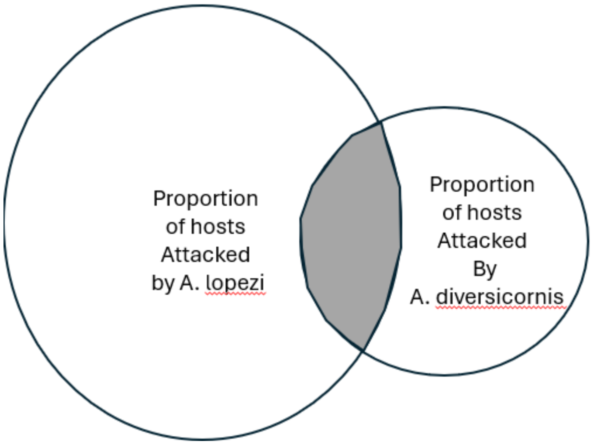

***A. diversicornis* life stages and parameters**

age := transit times for *A. diversicornus* in *dd* >13.5°C

(* age1----------------age2-------age3------------------------------age4

0 105 195 380dd

|--eggs -------larva-| --pupa-----| --------------------adults-------| *)

Potential hosts (${}^{Al}H$) for *A. diversicornis* weighted for *CM* stage preferences (i.e., stages [3-6]) plus preference of parasitoid egg-larval stage [1] resulting in multiple and super parasitism:

$${}^{Ad}H = 0.160\ CM[3] + 0.540\ CM[4] + 1.000\ CM[5] + 0.50\ CM[6] + 0.25\ Al + 0.25\ Ad,$$

and the demand for hosts is

$${}^{Ad}D = {}^{Ad}sr\,{}^{Al}\phi(T)[{}^{Ad}ovip(1+{}^{Ad}hf)\int {}^{Ad}adlt\ dx]^{Ad}\Delta x$$ with parameters

${}^{Ad}\phi(T) = (T,\ 13.5°C,\ 34°C)$, ${}^{Ad}ovip$ = 0.9/dd, and ${}^{Ad}sr$ = 0.5♀. ${}^{A1}\alpha$ = 0.6 is the search rate in eqn. 4.

Cassava green mite life stages and parameters

age := transit times for *CGM* in *dd* >14.65°C

```
(* cassava green mite
0                              86         98                             418dd
 |--------immatures--------|-preova-|--------adults ovip---------|
*)
```

Reproduction in CGM is computed as

$${}^{CGM}E = {}^{GM}sr\,{}^{CGM}\phi(T)\,{}^{CGM}(S/D)\sum_{1=1}^{k}1.8i/(1+1.15^{a})CM(i),$$

where ${}^{GM}sr$ = 0.8 female biased,

$0 \le {}^{CGM}\phi(T) = \left(-0.0164T^4 + 1.7381T^3 - 69.118T^2 + 1224.7T - 8104\right)/62.8 \le 1$ is the normalized left skewed effect of temperature [32],

$0 \le {}^{GM}\mu_{P^+} = 1.0 - e^{-0.0185 precip} < 1$ is the net rainfall/fungal mortality rate,

and $0.0 \le {}^{CGM}\mu(T) = (0.000012T^4 - 0.001187T^3 + 0.042353T^2 - 0.666530T + 3.938533) \le 1$ is the temperature-dependent mortality rate (e.g., text Fig. 8E) where $T = T_{mean}$.

***T. aripo* life stages and parameters**

age := transit times for *T. aripo* in *dd* > ${}^{Ta}\theta_L$ =11.4°C (from[43])

```
(* T. aripo
 0    28.6   42.2    58.48    74.8      110.2            350.0            396.0dd
 |-ova-|-larv-|-proto-|-deuto-|-preov-|--ovip-------|--post ovip --|
 0      1       2          3         4         5                  6                  7
*)
```

Predator age classes [1-7] μg demand for prey in prey egg equivalents/dd at 25°C.

^{Ta}Dem[1]:=0.0

^{Ta} Dem[2]:=0.24μg {micrograms per dd equals 9.4 prey eggs over the laval stage }

^{Ta} Dem[3]:=0.30μg { 15.6 eggs - protonymph stage}

^{Ta} Dem[4]:=0.56μg { 25 eggs - deutonymph stage}

^{Ta} Dem[5]:=0.54μg { 89 eggs - preoviposition female adult}

^{Ta} Dem[6]:=0.58μg {191 eggs over female oviposition period}

^{Ta} Dem[7]:=0.45μg { 95 eggs post-oviposition period}

^{Ta} fecundity:= 0.084 eggs/ *dd*> 11.4°C (Yaninek estimate 11/21/24 see [44])

^{Ta}sr= 0.66 {proportion females }

^{Ta}RHlim< 25.0% (limiting RH)

^{Ta}SD< 0.5 {pollen feeding begins and enables longer survival and reduced reproduction}

($^{Ta}\alpha$):= 1.2 {search rate}

$0 \leq {}^{Ta}\phi(T) = (T,\ 11.4°C,\ 35.4°C) \leq 1$ (temperature scalar with limits 11.4-35.4°C; ref. [44])

$^{Ta}\mu(RH)$ = min(0.99, 1-$exp^{-2.0/(RHmean-RHlim)}$) is desiccation mortality rate where RHlim= 10%.

***A. manihoti* life stages and parameters**

age := transit times for *A. manihoti* in *dd* > $^{Aml}\theta_L$ =6.64°C

```
(*
A. manihoti substage age intervals in degree days >  6.64°C)
    0    39.06  61.4    81.8      98.6     120.9          254.8              288.30
    |-ova-|-larv-|-proto-|-deuto-|-preov-|--ovip-------|--post ovip --|
    0       1         2           3          4           5                   6                     7
*)
```

Predator age classes [1-7] μg demand for prey in prey egg equivalents/dd at 25°C{it attacks its own)}

^{Am}Dem[1] = 0.0

^{Am}Dem[2] = 0.22μg {1 μg per dd}{ 16.0 eggs - laval stage }

^{Am}Dem[3] = 0.21μg {18 eggs - protonymph stage }

^{Am}mDem[4] = 0.26μg {23 eggs - deutonymph stage }

^{Am}Dem[5] = 0.41μg {98 eggs - preoviposition }

^{Am}Dem[6] = 0.37μg {257 - oviposition period }

^{Am}Dem[7] = 0.27μg {63 - post-oviposition at 25°C}

^{Am}fecundity = 0.18 eggs/dd>6.64°C

^{Am}sr = 0.66 { proportion females }

^{Am}RHlim = 40%

$0 \leq {}^{Am}SD_{min} \leq 1$ {no pollen effect for *A. manihoti*}

$^{Am}\alpha =$ 0.8 {search rate < random}

$^{Am}\phi(T) = (T,\ 6.64°C,\ 32.8°C)$ (temperature scalar with limits 6.64 - 32.8°C

Desiccation mortality where VPD is vapor pressure deficit (see[62])

$^{Am}\mu(T,Rh)$ = max (0, min {0.99, 1.0 - 0.1658 VPD + 1.1054}) where

$$VPD = 610.7 \cdot (10^{7.5T/(237.3+T)})(1 - Rh/100)/1000,$$

and *T* is mean temperature and Rh is % relative humidity.

Distributed maturation time population dynamics model

The models used herein are time varying life tables (TVLT), the parameter of which may be MP or BDF based (see above). Suitable population dynamics models used to capture the dynamics of interacting species were reviewed by Gutierrez [61], Di Cola *et al.* [63], and Buffoni and Pasquali [64]. For simplicity, we use the discrete form of the time-invariant distributed-maturation time demographic models [65,66] parameterized using the MP and/or BDF biology. The time-varying form of the model [67] is appropriate where the developmental time of the species and substages change over time in response to various factors (e.g., nutrition).

In the model, the dynamics of a life stage *s* of average developmental time ${}^s\Delta$ having i=1, 2,...,${}^sk$ age classes can be viewed as composed of ${}^sk$ dynamics equations [61,68] (eqn. 5). Using the notation of Di Cola *et al.* (page 523) [63], the ith age class of stage *s* is modeled as follows:

$$\frac{d\,{}^sN_i}{dt} = \frac{{}^sk \cdot {}^s\Delta x}{{}^s\Delta}\left[{}^sN_{i-1}(t) - {}^sN_i(t)\right] - {}^s\mu_i(t)\,{}^sN_i(t)\,. \qquad \text{[A1i]}$$

In terms of flux, ${}^sn_i(t) = {}^sN_i(t)\,{}^s\nu_i(t)$ where ${}^s\nu_i(t) = \frac{{}^sk}{{}^s\Delta}\Delta x(t)$, and

$$\frac{d}{dt}\left[\frac{{}^s\Delta\,{}^sn_i(t)}{{}^sk}\right] = {}^sn_{i-1}(t) - {}^sn_i(t) - {}^s\mu_i(t)\,{}^sn_i(t)\frac{{}^s\Delta}{{}^sk}\,. \qquad \text{[A1ii]}$$

Absent mortality, the theoretical distribution of cohort developmental times of stage *s* may be estimated by Erlang parameter ${}^sk = {}^s\Delta^2 / {}^s\sigma^2$, where σ^2 is the variance of ${}^s\Delta$ (i.e., theoretically, a cohort entering a stage will have Erlang distributed maturation times centered on ${}^s\Delta$). Appropriate data were unavailable, and hence a value of k = 50 was used for all the species.

The forcing variable is temperature (*T*), with chronological time (*t*) in days (*d*) that from the perspective of poikilotherm species is of variable length in physiological time units (i.e., $0 \leq {}^s\Delta x(T(t))$ in degree days (*dd*)), or proportional development (${}^sr(T(t))$). The state variable ${}^sN_i(t)$ is the density of the ith age class (mass or numbers), and ${}^s\mu_i(t)$ is the proportional age-specific net loss rate (losses and gains) due to temperature, net immigration, growth in mass dynamics models, and other factors during ${}^s\Delta x(T(t))$[61]. For computational efficiency in the model, the components of ${}^s\mu_i(t)$ are computed and applied after aging. The total density of a life stage *s* is ${}^sN(t) = \sum_{i=1}^{k} {}^sN_i(t)$. New individuals enter the first age class of a stage 1, flows occur via aging between age classes and between stages, and surviving adults exit as deaths from maximum age (i = ${}^Ak$).

Variations of ${}^s\mu_i(t)$ in this model easily facilitate capturing the biology of any species. The numerical solution for eqn. A1ii is found in Abkin and Wolf [65], and as implemented here in Gutierrez (pages 157-159) [61].

References for Supplemental Materials

1. Capra, F. & Luisi, P. L. *The Systems View of Life: A Unifying Vision*. (Cambridge university press, Cambridge, 2014).
2. Löhr, B., Neuenschwander, P., Varela, A. M. & Santos, B. Interactions between the female parasitoid *Epidinocarsis lopezi* De Santis (Hym., Encyrtidae) and its host, the cassava mealybug, *Phenacoccus manihoti* Matile-Ferrero (Hom., Pseudococcidae). *J Applied Entomology* **105**, 403–413 (1988).
3. Neuenschwander, P. & Madojemu, E. Mortality of the cassava mealybug, *Phenacoccus manihoti* Mat.-Ferr. (Hom., Pseudococcidae), associated with an attack by *Epidinocarsis lopezi* (Hym., Encyrtidae). (1986) doi:10.5169/SEALS-402203.
4. Neuenschwander, P., Schulthess, F. & Madojemu, E. Experimental evaluation of the efficiency of *Epidinocarsis lopezi* , a parasitoid introduced into Africa against the cassava mealybug *Phenacoccus manihoti*. *Entomologia Exp Applicata* **42**, 133–138 (1986).
5. Boussienguet, J., Neuenschwander, P. & Herren, H. R. Le complexe entomophage de la cochenille du manioc au Gabon. 4. Etablissement du parasitoide *Epidinocarsis lopezi*. *Entomophaga* **36**, 455–469 (1991).
6. Chakupurakal, J. *et al.* Biological Control of the Cassava Mealybug, *Phenacoccus manihoti* (Homoptera: Pseudococcidae), in Zambia. *Biological Control* **4**, 254–262 (1994).
7. Neuenschwander, P., Hammond, W. N. O. & Hennessey, R. D. Changes in the composition of the fauna associated with the cassava mealybug, *Phenacoccus manihoti*, following the introduction of the parasitoid *Epidinocarsis lopezi*. *Insect Science and its Application* **8**, 893–898 (1987).
8. Neuenschwander, P., Hennessey, R. D. & Herren, H. R. Food web of insects associated with the cassava mealybug, *Phenacoccus manihoti* Matile-Ferrero (Hemiptera: Pseudococcidae), and its introduced parasitoid, *Epidinocarsis lopezi* (De Santis) (Hymenoptera: Encyrtidae), in Africa. *Bull. Entomol. Res.* **77**, 177–189 (1987).
9. Schulthess, F., Neuenschwander, P. & Gounou, S. Multi-trophic interactions in cassava, <i>Manihot esculenta<i>, cropping systems in the subhumid tropics of West Africa. *Agriculture, Ecosystems & Environment* **66**, 211–222 (1997).
10. Goergen, G. & Neuenschwander, P. A cage experiment with four trophic levels: cassava plant growth as influenced by cassava mealybug, *Phenacoccus manihoti*, its parasitoid *Epidinocarsis lopezi*, and the hyperparasitoids *Prochiloneurus insolitus* and *Chartocerus hyalipennis*. *Journal of Plant Diseases and Protection* **99**, 182–190 (1992).
11. Neuenschwander, P. & Hammond, W. N. O. Natural enemy activity following the introduction of <i>Epidinocarsis lopezi<i> (Hymenoptera: Encyrtidae) against the cassava mealybug., *Phenacoccus*

manihoti (Homoptera: Pseudococcidae)., in southwestern Nigeria. *Environmental Entomology* **17**, 894–902 (1988).

12. Neuenschwander, P. & Sullivan, D. Interactions between the endophagous parasitoid *Epidinocarsis lopezi* and its host, *Phenacoccus manihoti*. *Int J Trop Insect Sci* **8**, 857–859 (1987).
13. Stäubli Dreyer, B., Baumgärtner, J. & Neuenschwander, P. The functional responses of two *Hyperaspis notata* strains to their prey, the cassava mealybug *Phenacoccus manihoti*. (1997) doi:10.5169/SEALS-402652.
14. Stäubli Dreyer, B., Neuenschwander, P., Baumgärtner, J. & Dorn, S. Trophic influences on survival, development and reproduction of *Hyperaspis notata* (Col., Coccinellidae). *J Applied Entomology* **121**, 249–256 (1997).
15. Gutierrez, A. P., Neuenschwander, P. & Alphen van, J. J. M. Factors affecting biological control of cassava mealybug by exotic parasitoids: a ratio-dependent supply-demand driven model. *Journal of Applied Ecology* **30**, 706–721 (1993).
16. Pijls, J. W. a. M., Hofker, K. D., Staalduinen, M. J. V. & Alphen, J. J. M. V. Interspecific host discrimination and competition in *Apoanagyrus (Epidinocarsis) lopezi* and *A.(E.)diversicornis*, parasitoids of the cassava mealybug *Phenacoccus manihoti*. *Ecological Entomology* **20**, 326–332 (1995).
17. Cudjoe, A. R., Neuenschwander, P. & Copland, M. J. W. Experimental determination of the efficiency of indigenous and exotic natural enemies of the cassava mealybug, *Phenacoccus manihoti* Mat.-Ferr. (Hom., Pseudococcidae), in Ghana. *Journal of Applied Entomology* **114**, 77–82 (1992).
18. Cudjoe, A. R., Neuenschwander, P. & Copland, M. J. W. Interference by ants in biological control of the cassava mealybug *Phenacoccus manihoti* (Hemiptera: Pseudococcidae) in Ghana. *Bull. Entomol. Res.* **83**, 15–22 (1993).
19. Wyckhuys, K. A. G. *et al.* Continental-scale suppression of an invasive pest by a host-specific parasitoid underlines both environmental and economic benefits of arthropod biological control. *PeerJ* **6**, e5796 (2018).
20. Gutierrez, A. P., Yaninek, J. S., Wermelinger, B., Herren, H. R. & Ellis, C. K. Analysis of biological control of cassava pests in Africa. III. Cassava green mite *Mononychellus tanajoa*. *J. Appl. Ecol.* **25**, 941–950 (1988).
21. Gutierrez, A. P., Yaninek, J. S., Neuenschwander, P. & Ellis, C. K. A physiologically-based tritrophic metapopulation model of the African cassava food web. *Ecological Modelling* **123**, 225–242 (1999).
22. Yaseen, M. & Bennett, F. D. Distribution, biology, and population dynamics of the green cassava mite in the neotropics. in *Proceedings of the Fourth Symposium of the International Society for Tropical*

Root Crops, 1-7 August 1976, Cali, Colombia (eds Cock, J., Maclntyre, R. & Graham, M.) 197–202 (IDRC, Ottawa, Canada, 1977).

23. Bellotti, A. & Schoonhoven, A. van. Mite and insect pests of cassava. *Annual Review of Entomology* **23**, 39–67 (1978).
24. Samways, M. J. Immigration, population growth and mortality of insects and mites on cassava in Brazil. *Bulletin of Entomological Research* **69**, 491–505 (1979).
25. Elliot, S. L. *et al.* Potential of the mite-pathogenic fungus *Neozygites floridana* (Entomophthorales: Neozygitaceae) for control of the cassava green mite *Mononychellus tanajoa* (Acari: Tetranychidae). *Bulletin of Entomological Research* **90**, 191–200 (2000).
26. Yaninek, J. S., Saizonou, S., Onzo, A., Zannou, I. & Gnanvossou, D. Seasonal and habitat variability in the fungal pathogens, *Neozygites* cf. *floridana* and *Hirsutella thompsonii*, associated with cassava mites in Benin, West Africa. *Biocontrol Science and Technology* **6**, 23–34 (1996).
27. Lyon, W. F. A green cassava mite recently found in Africa. *Plant Prot. Bull.* **22**, 11–13 (1974).
28. Nyiira, Z. Advances in research on the economic significance of the green cassava mite (*Mononychellus tanajoa*) in Uganda. in *The International Exchange and Testing of Cassava Germ Plasm in Africa. Proceedings of an interdisciplinary workshop held at IITA, Ibadan, Nigeria 17-21 November 1975* (eds Terry, E. & MacIntyre, R.) 27–29 (International Development Research Centre, Ottawa, Canada, 1976).
29. Shukla, P. Preliminary report on the green mite (*Mononychellus tanajoa*, Bonder) resistance in Tanzanian local cassava varieties. *East African Agricultural and Forestry Journal* **42**, 55–59 (1976).
30. Ndayiragije, P. Cassava green mite (*Mononychellus tanajoa* (Bondar)) in Burundi. *Integrated pest management of cassava green mite. Proceedings of a regional training workshop in East Africa, 30 April - 4 May 1984* 67–73 (1984) doi:10.5555/19850521480.
31. Yaninek, J. S., Gutierrez, A. P. & Herren, H. R. Dynamics of *Mononychellus tanajoa* (Acari: Tetranychidae) in Africa: effects on dry matter production and allocation in cassava. *Environmental Entomology* **19**, 1767–1772 (1990).
32. Yaninek, J. S., Gutierrez, A. P. & Herren, H. R. Dynamics of *Mononychellus tanajoa* (Acari: Tetranychidae) in Africa: experimental evidence of temperature and host plant effects on population growth rates. *Environmental Entomology* **18**, 633–640 (1989).
33. Yaninek, J. S., Herren, H. R. & Gutierrez, A. P. Dynamics of *Mononychellus tanajoa* (Acari: Tetranychidae) in Africa: seasonal factors affecting phenology and abundance. *Environmental Entomology* **18**, 625–632 (1989).

34. Moraes, G. J. D., Moreira, A. N. & Delalibera, I. Growth of the mite *Mononychellus tanajoa* (Acari: Tetranychidae) on alternative plant hosts in northeastern Brazil. *Florida Entomologist* **78**, 350–354 (1995).

35. Yaninek, J. S. & Bellotti, A. C. Exploration for natural enemies of cassava green mites based on agrometeorological criteria. in *Proceedings of the Seminar on Agrometeorology and Crop Protection in the Lowland Humid and Subhumid Tropics, Cotonou, Benin, 7-11 July 1986* (eds Rijks, D. & Mathys, G.) 69–75 (World Meteorological Organization, Geneva, Switzerland, 1987).

36. Onzo, A., Hanna, R., Zannou, I., Sabelis, M. W. & Yaninek, J. S. Dynamics of refuge use: diurnal, vertical migration by predatory and herbivorous mites within cassava plants. *Oikos* **101**, 59–69 (2003).

37. Gnanvossou, D., Hanna, R., Dicke, M. & Yaninek, S. J. Attraction of the predatory mites *Typhlodromalus manihoti* and *Typhlodromalus aripo* to cassava plants infested by cassava green mite. *Entomologia Experimentalis et Applicata* **101**, 291–298 (2001).

38. Onzo, A., Sabelis, M. W. & Hanna, R. Effects of ultraviolet radiation on predatory mites and the role of refuges in plant structures. *Environmental Entomology* **39**, 695–701 (2010).

39. Gnanvossou, D., Hanna, R. & Dicke, M. Infochemical-mediated niche use by the predatory mites *Typhlodromalus manihoti* and *T. aripo* (Acari: Phytoseiidae). *Journal of Insect Behavior* **16**, 523–535 (2003).

40. Magalhães, S., Brommer, J. E., Silva, E. S., Bakker, F. M. & Sabelis, M. W. Life-history trade-off in two predator species sharing the same prey: a study on cassava-inhabiting mites. *Oikos* **102**, 533–542 (2003).

41. Gnanvossou, D., Hanna, R., Yaninek, J. S. & Toko, M. Comparative life history traits of three neotropical phytoseiid mites maintained on plant-based diets. *Biological Control* **35**, 32–39 (2005).

42. Cuellar, M. E., Calatayud, P.-A., Melo, E. L., Smith, L. & Bellotti, A. C. Consumption and oviposition rates of six phytoseiid species feeding on eggs of the cassava green mite *Mononychellus tanajoa* (Acari: Tetranychidae). *The Florida Entomologist* **84**, 602–607 (2001).

43. Gnanvossou, D., Yaninek, J. S., Hanna, R. & Dicke, M. Effects of prey mite species on life history of the phytoseiid predators *Typhlodromalus manihoti* and *Typhlodromalus aripo*. *Exp Appl Acarol* **30**, 265–278 (2003).

44. Mutisya, D. L., El-Banhawy, E. M., Kariuki, C. W. & Khamala, C. P. M. *Typhlodromalus aripo* De Leon (Acari: Phytoseiidae) development and reproduction on major cassava pests at different temperatures and humidities: an indication of enhanced mite resilience. *Acarologia* **54**, 395–407 (2014).

45. Bonato, O., Da S. Noronha, A. C. & De Moraes, G. Distribution et échantillonnage des populations de *Amblyseius manihoti* Moraes (Acari, Phytoseiidae) sur manioc au Brésil. *Journal of Applied Entomology* **123**, 541–546 (1999).
46. Yaninek, J. S. *et al.* Establishment and spread of *Typhlodromalus manihoti* (Acari: Phytoseiidae), an introduced phytoseiid predator of *Mononychellus tanajoa* (Acari: Tetranychidae) in Africa. *Environmental Entomology* **27**, 1496–1505 (1998).
47. Gutierrez, A. P. *et al.* Analysis of biological control of cassava pests in Africa. I. Simulation of carbon, nitrogen and water dynamics in cassava. *Journal of Applied Ecology* **25**, 901–920 (1988).
48. Gutierrez, A. P. *et al.* Analysis of biological control of cassava pests in Africa. II. Cassava mealybug *Phenacoccus manihoti*. *J. Appl. Ecol.* **25**, 921–940 (1988).
49. de Wit, C. T. & Goudriaan, J. *Simulation of Ecological Processes*. vol. 2nd (Pudoc, Wageningen, The Netherlands, 1978).
50. Gutierrez, A. P. & Baumgärtner, J. U. Multitrophic level models of predator-prey energetics: I. Age-specific energetics models - pea aphid *Acyrthosiphon pisum* (Homoptera: Aphididae) as an example. *Can. Entomol.* **116**, 924–932 (1984).
51. Gilbert, N., Gutierrez, A. P., Frazer, B. D. & Jones, R. E. *Ecological Relationships*. (W.H. Freeman and Co., Reading and San Francisco, 1976).
52. Gutierrez, A. P. & Ponti, L. Eradication of invasive species: why the biology matters. *Environ. Entomol.* **42**, 395–411 (2013).
53. Gutierrez, A. P., Ponti, L., Neteler, M., Suckling, D. M. & Cure, J. R. Invasive potential of tropical fruit flies in temperate regions under climate change. *Commun Biol* **4**, 1–14 (2021).
54. Maurer, V. & Baumgärtner, J. Temperature influence on life table statistics of the chicken mite *Dermanyssus gallinae* (Acari: Dermanyssidae). *Exp Appl Acarol* **15**, 27–40 (1992).
55. Maurer, V. & Baumgärtner, J. A population model for *Dermanyssus gallinae* (Acari: Dermanyssidae). *Exp Appl Acarol* **18**, 409–422 (1994).
56. Fouque, F., Baumgärtner, J. & Delucchi, V. Analysis of temperature-dependent stage-frequency data of *Aedes vexans* (MEIGEN) populations originated from the Magadino plain (Southern Switzerland). *Bulletin of the Society for Vector Ecology* **17**, 28–38 (1992).
57. Fouque, F. & Baumgärtner, J. Simulating development and survival of *Aedes vexans* (Diptera: Culicidae) preimaginal stages under field conditions. *Journal of Medical Entomology* **33**, 32–38 (1996).
58. Dreyer, H. & Baumgärtner, J. The influence of post-flowering pests on cowpea seed yield with particular reference to damage by Heteroptera in southern Benin. *Agriculture, Ecosystems & Environment* **53**, 137–149 (1995).

59. Keating, B. A. & Evenson, J. P. Effect of soil temperature on sprouting and sprout elongation of stem cuttings of cassava (*Manihot esculenta* Crantz.). *Field Crops Research* **2**, 241–251 (1979).
60. Campbell, A., Frazer, B. D., Gilbert, N., Gutierrez, A. P. & Mackauer, M. Temperature requirements of some aphids and their parasites. *Journal of Applied Ecology* **11**, 431–438 (1974).
61. Gutierrez, A. P. *Applied Population Ecology: A Supply-Demand Approach*. (John Wiley and Sons, New York, USA, 1996).
62. Mégevand, B. Some factors affecting the establishment of exotic phytoseiids in a new environment. 111 S. (Swiss Federal Institute of Technology Zurich, 1997). doi:10.3929/ETHZ-A-001817698.
63. Di Cola, G., Gilioli, G. & Baumgärtner, J. Mathematical models for age-structured population dynamics. in *Ecological entomology* (eds Huffaker, C. B. & Gutierrez, A. P.) (Wiley, New York, USA, 1999).
64. Buffoni, G. & Pasquali, S. Structured population dynamics: continuous size and discontinuous stage structures. *J. Math. Biol.* **54**, 555–595 (2007).
65. Abkin, M. H. & Wolf, C. *Computer Library for Agricultural Systems Simulation. Distributed Delay Routines: DEL, DELS, DELF, DELLF, DELVF, DELLVF*. (Department of Agricultural Economics, Michigan State University, Lansing, MI, USA, 1976).
66. Manetsch, T. J. Time-varying distributed delays and their use in aggregative models of large systems. *IEEE transactions on Systems, Man and Cybernetics* **6**, 547–553 (1976).
67. Vansickle, J. Attrition in distributed delay models. *IEEE T. Syst. Man Cyb.* **7**, 635–638 (1977).
68. Severini, M., Alilla, R., Pesolillo, S. & Baumgärtner, J. Fenologia della vite e della *Lobesia botrana* (Lep. Tortricidae) nella zona dei Castelli Romani. *Rivista Italiana di Agrometeorologia* **3**, 34–39 (2005).
